# Safety and Efficacy of Pulse Field Ablation in the Treatment of Atrial Fibrillation and Its Comparison with Traditional Thermal Ablation: A Systematic Review and Meta-Analysis

**DOI:** 10.31083/j.rcm2511415

**Published:** 2024-11-21

**Authors:** Aobo Gong, Wentao Li, Fanghui Li, Yao Tong, Ying Cao, Rui Zeng

**Affiliations:** ^1^Department of Cardiology, West China Hospital, Sichuan University, 610041 Chengdu, Sichuan, China

**Keywords:** atrial fibrillation, pulsed field ablation, thermal ablation, systematic review, meta-analysis

## Abstract

**Background:**

The purpose of this meta-analysis was to evaluate the efficacy and safety of pulsed field ablation (PFA) and to compare it with the efficacy and safety of traditional thermal ablation in patients with atrial fibrillation (AF).

**Methods:**

PubMed, Web of Science, and Embase were searched for randomized or observational studies exploring the efficacy and safety of PFA and comparing PFA with traditional thermal ablation in patients with AF.

**Results:**

A total of 4437 patients from 16 studies that only included PFA as the ablation method and 1792 patients from 9 comparing PFA to traditional thermal ablation were included in the final analysis. In studies that considered PFA alone, the freedom from atrial arrhythmia recurrence was 0.80 (95% confidence interval [CI] 0.76–0.84), and the incidence of periprocedural complications was 0.03 (95% CI 0.02–0.05). In comparative studies, there was no significant difference in the freedom from atrial arrhythmia recurrence (odds ratio (OR) 1.24, 95% CI 0.90–1.72) and the incidence of periprocedural complications (OR 0.74, 95% CI 0.37–1.48) of PFA compared to that of traditional thermal ablation. In the subgroup with a follow-up period less than 12 months, PFA had higher freedom from atrial arrhythmia recurrence rate compared to thermal ablation (OR 2.19, 95% CI 1.14–4.20).

**Conclusions:**

PFA is a safe and effective catheter ablation method that is not inferior to the traditional and well-established thermal ablation. It can be used as a treatment of choice for patients with AF.

**The PROSPERO registration:**

CRD42023473026, https://www.crd.york.ac.uk/PROSPERO/display_record.php?RecordID=473026.

## 1. Introduction

Catheter ablation is recommended for long-term rhythm control in patients with atrial fibrillation (AF) [1]. Traditional thermal ablation refers to the application of radiofrequency or cryoenergy to heat or freeze tissue, resulting in localized tissue necrosis, and is the most well-established energy source for catheter ablation in clinical practice [2]. However, in previous clinical practice and studies, there were still some non-negligible complications caused by traditional thermal ablation, which caused significant postoperative discomfort and even led to pulmonary vein stenosis, persistent phrenic nerve paralysis, atrial esophageal fistula, and even death [3, 4]. Moreover, thermal ablation has a high learning cost [5], but its ability to achieve long-term arrhythmia freedom has not met expectations [6].

Unlike thermal ablation, pulsed field ablation (PFA) is a novel method that utilizes short-duration and high-voltage electricity to induce electroporation [7, 8]. Various tissues and cell types have different characteristic threshold pulsed-field strengths, and cardiomyocytes have the lowest threshold values of any tissue [9]. Therefore, with appropriate settings, we can selectively kill only myocardial cells without affecting other tissues, thereby reducing the occurrence of other complications and improving the safety of the procedure [7]. Furthermore, PFA can reduce the procedure time to improve patient comfort during and after the procedure and achieve myocardial cell death simultaneously [10]. Therefore, PFA, as a method of myocardial-specific ablation, appears to have the ability to overcome the limitations of traditional catheter ablation.

Since 2018, many studies have suggested that PFA may be an effective and safe ablation option for AF [9, 11, 12]. However, the quality of the articles published on the efficacy and safety of PFA varies and has not been effectively integrated, making it difficult to guide clinical practice. Moreover, the advantages of PFA over traditional thermal ablation have not been fully elaborated. Therefore, a meta-analysis of existing studies is necessary to provide evidence-based support to assist clinicians in making proper decisions. Hence, we conducted a systematic review and meta-analysis to evaluate the safety and efficacy of PFA and compare them with traditional thermal ablation for AF.

## 2. Materials and Methods

The methods and results of this study were performed according to the Preferred Reporting Items for Systematic Reviews and Meta-analyses statement (PRISMA) (**Supplementary Table 1**). The registration number is CRD42023473026. The research protocol was revised to provide a more comprehensive evaluation of the PFA.

### 2.1 Search Strategy

Data from published trials were obtained by searching PubMed, Web of Science, and Embase databases from database inception through the final search date of October 19, 2023. We considered the following keywords to search for relevant studies: “atrial fibrillation”, “pulsed-field ablation”, “electroporation”, and “pulsed electric field ablation”. We restricted the search to human studies and clinical trials. Further, literature retrieval was performed by screening the reference lists of the included articles. The search formula is presented in **Supplementary Table 2**.

### 2.2 Study Selection

For the studies that only included PFA as the ablation method, the inclusion criteria were studies on patients with AF receiving PFA, regardless of whether the type of AF was paroxysmal or persistent (including long-standing persistent AF). The efficacy outcome was freedom from atrial arrhythmia recurrence (atrial tachycardia, atrial flutter, or AF). The recurrence of atrial arrhythmia was detected by electrocardiography. The safety endpoint is the incidence of PFA system-related or PFA procedure-related adverse events during the perioperative period. For the only-PFA studies, we excluded duplicate studies and studies without a full text, at least 3-month follow-up, the endpoints of interest, or quality score >6 points according to the Methodological Index for Non-Randomized Studies (MINORS) criteria [13].

For studies comparing PFA to traditional thermal ablation, the inclusion criteria, efficacy endpoints and safety endpoints were the same as those in only-PFA studies. Duplicate studies and articles without clear follow-up durations or the endpoints of interest were excluded.

### 2.3 Data Collection and Quality Assessment

Baseline characteristics, follow-up design, data on efficacy and safety endpoints, characteristics of the PFA and thermal ablation systems, and procedure methods were independently extracted by two investigators. Disagreements were resolved through consensus or consultation with a third reviewer. To ensure consistency in reviewing and reporting the results, two reviewers independently assessed methodological quality. Studies included only PFA as the ablation method used the MINORS criteria [13]. For studies comparing PFA to traditional thermal ablation, the reviewers chose the Cochrane risk of bias tool or Newcastle–Ottawa Scale (NOS) criteria based on their article type.

### 2.4 Statistical Analysis 

The STATA software (version 17.0, Stata Corp., College Station, TX, USA) was used for all data analyses. The odds ratio (OR) and 95% confidence intervals (CI) were calculated by random-effects models to pool the effects obtained in each study. I^2^ statistics (>50% was considered significant heterogeneity) were performed to identify the cause of heterogeneity. We used the Funnel plots to evaluate potential publication bias. Additionally, we conducted a sensitivity analysis for the primary results. Subgroup analyses were conducted according to the type of AF, follow-up duration, and ablation strategy in order to reduce potential heterogeneity.

## 3. Results

### 3.1 Literature Search, Study Selection, and Included Study Characteristics

**Fig. 1.**
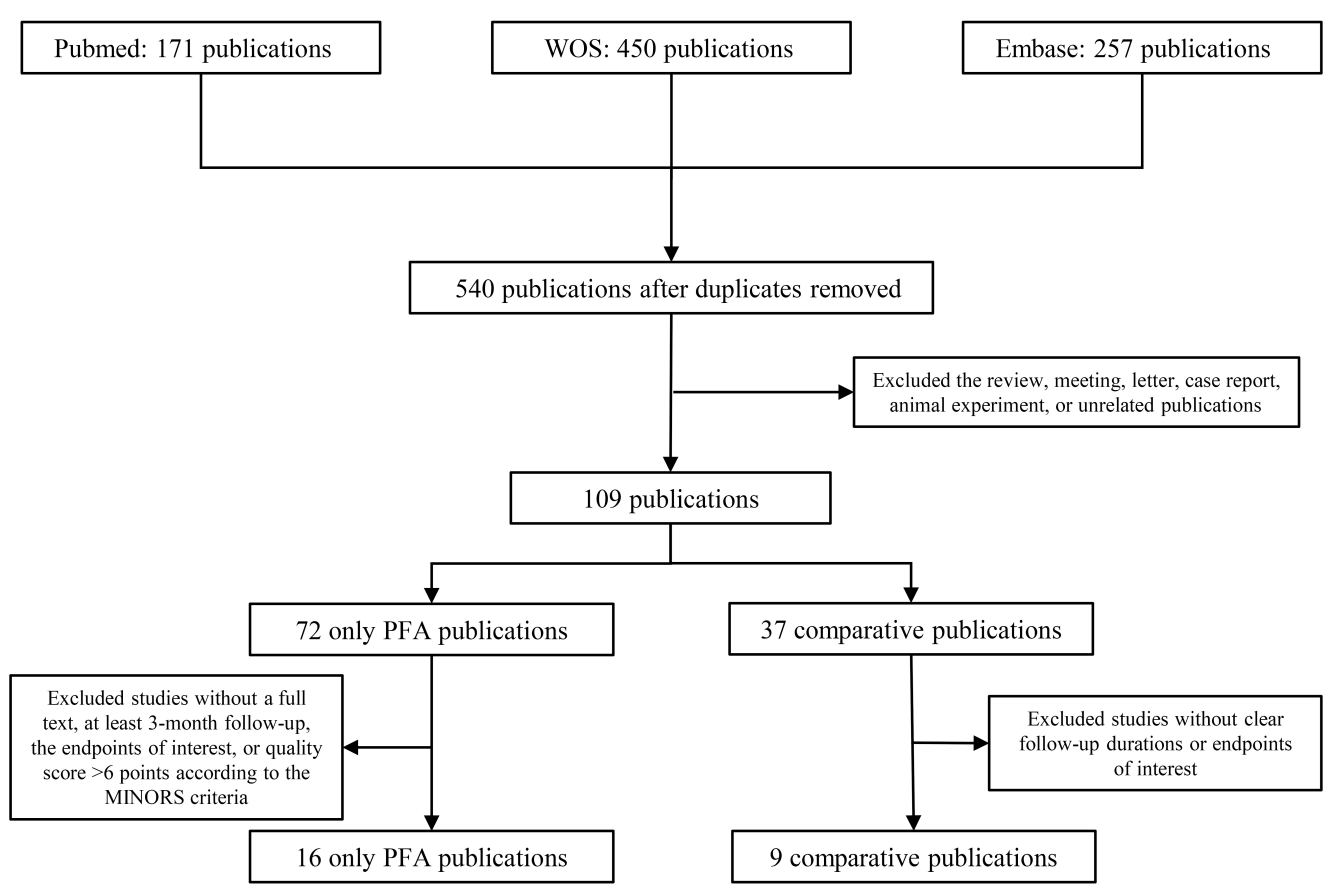
**Flow chart**. Abbreviation: WOS, web of science; PFA, pulsed field ablation; MINORS, Methodological Index for Non-Randomized Studies.

Searching the PubMed, Web of Science, and Embase databases from their inception until the final search date and excluding duplicate studies, 540 studies remained. After excluding reviews, meetings, letters, case reports, animal experiments, and unrelated publications, 109 articles remained. After applying the exclusion criteria for studies that included only PFA as the ablation method and those comparing PFA with thermal ablation, 25 studies were remaining. The detailed quality assessment results are presented in Table 1 (Ref. [5, 14, 15, 16, 17, 18, 19, 20]), **Supplementary Table 3** and **Supplementary Fig. 1**. The remaining 16 only-PFA publications [9, 11, 12, 22, 23, 24, 25, 26, 27, 28, 29, 30, 31, 32, 33, 34] and 9 comparative publications [5, 14, 15, 16, 17, 18, 19, 20, 21] were included in this meta-analysis (Fig. 1).

**Table 1.** **The quality assessment of ten comparative studies between pulsed field ablation and traditional thermal ablation according to the Newcastle-Ottawa Scale criteria**.

Author	Representativeness of the exposed cohort	Selection of the nonexposed cohort	Ascertainment of exposure	Demonstration that outcome of interest was not present at start of study	Comparability of cohorts on the basis of the design or analysis	Assessment of outcome	Was follow-up long enough for outcomes to occur	Adequacy of follow up of cohorts	NOS score
Urbanek *et al*. [14] (2023)	★	★	★	★	★★	★	★	★	9
Maurhofer *et al*. [15] (2023)	★	✩	★	★	★	★	★	★	7
Weidlich *et al*. [16] (2023)	★	★	★	✩	✩	★	★	★	6
Nakatani *et al*. [17] (2021)	★	★	★	★	★★	★	★	★	9
Kupusovic *et al*. [18] (2023)	★	★	★	★	★★	★	★	★	9
Schipper *et al*. [5] (2023)	★	✩	★	★	★★	★	★	★	8
Woermann *et al*. [19] (2023)	★	★	★	✩	✩	★	★	★	6
Kueffer *et al*. [20] (2023)	★	✩	★	✩	★★	★	★	★	7

Note: ★indicates 1 score, ✩indicates 0 score. All included comparative studies have a NOS score of 6 or higher. Abbreviation: NOS, Newcastle Ottawa Scale.

A summary of the 16 only-PFA studies is presented in **Supplementary Table 4**. The 9 comparative studies (Table 2, Ref. [5, 14, 15, 16, 17, 18, 19, 20, 21]) included 1792 patients. The participants were middle-aged and older individuals. Patients with paroxysmal, persistent, or long-standing persistent AF were included. All PFA systems and ablation procedures were similar.

**Table 2.** **Summary of the characteristics, procedures, and outcomes for all included comparative studies of thermal ablation and pulsed field ablation**.

Study	Study type	Group size	AF pattern	Follow-up	Energy	Total procedure duration	Fluoroscopy time	Recurrence	Total complications
Reddy *et al*. [21] (2023)	RCT	305	PAF: 607 (100%)	12-month	PFA	105.8 ± 29.4	21.1 ± 11.0	51 (17.2%)	7 (2.3%)
		302			RFA and CBA	123.1 ± 42.1	13.9 ± 12.8	48 (16.4%)	6 (2%)
Urbanek *et al*. [14] (2023)	Retrospective cohert study	200	PAF: 116 (58%)	12-month	PFA	34.5 (29–40)	7.1 (5.5–8.9)	52 (26%)	6 (3%)
		200	PAF: 127 (63.5%)		CBA	50 (45–60)	6.9 (5.5–8.8)	49 (24.5%)	13 (6.5%)
Maurhofer *et al*. [15] (2023)	Prospective cohert study	40	PAF: 200 (100%)	12-month	PFA	93.5 (79.5–116)	25.6 (20.7–31)	6 (15%)	2 (5%)
		160	PAF: 9 (60%)		RFA and CBA	CBA: 75 (60–97), RFA: 182 (134.2–223.5)	CBA: 17.1 (12.7–23.7), RFA: 6.7 (3.5–12.9)	48 (30%)	0
Kupusovic *et al*. [18] (2023)	Retrospective cohert study	15		6-month	PFA	179.3 ± 49.3	31.1 ± 9.8	0	0
		11	PAF: 4 (36.4%)		CBA	177.3 ± 56.5	23.1 ± 7.0	1 (9.1%)	0
Schipper *et al*. [5] (2023)	Retrospective cohert study	54	PAF: 16 (30%)	12-month	PFA	64.5 ± 17.5	15.3 ± 4.7	12 (26%)	2 (3.7%)
		54	PAF: 17 (31%)		CBA	73.0 ± 24.8	12.3 ± 5.3	14 (28%)	6 (11.1%)
Nakatani *et al*. [17] (2021)	Prospective cohert study	18	PAF: 41 (100%)	9-month	PFA	96 (77–111)	23 (17–29)	2 (11.1%)	1 (5.6%)
		23			RFA and CBA	130 (110–200)	20 (18–31)	9 (39.1%)	2 (8.7%)
Woermann *et al*. [19] (2023)	Cohert study	57	PAF: 17 (30%)	3-month	PFA	65 ± 17	15 ± 5	11 (19.3%)	2 (3.5%)
		57	-		RFA	95 ± 23	12 ± 3	13 (22.8%)	3 (5.3%)
Weidlich *et al*. [16] (2023)	Cohert study	56	PAF: 65 (55%)	6-month	PFA	58 (51–70)	12 (10–16)	5 (9%)	1 (1.8%)
		63			RFA	83 (71–99)	2.2 (1.3–3.6)	15 (24%)	1 (1.6%)
Kueffer *et al*. [20] (2023)	Cohert study	65	PSAF	12-month	PFA	109 (88–130)	26 (19–31)	44%	0
		112			RFA and CBA	CBA: 81 (62–96), RFA: 177 (153–200)	CBA: 18 (15–24), RFA: 7 (3–14)	CBA: 33%, RFA: 51%	0

Abbreviation: AF, atrial fibrillation; PFA, pulse field ablation; RFA, radiofrequency ablation; CBA, cryoballoon ablation; RCT, randomized controlled trial; PAF, paroxysmal atrial fibrillation; PSAF, persistent atrial fibrillation.

### 3.2 Pooled Estimates of Efficacy and Safety of PFA

**Fig. 2.**
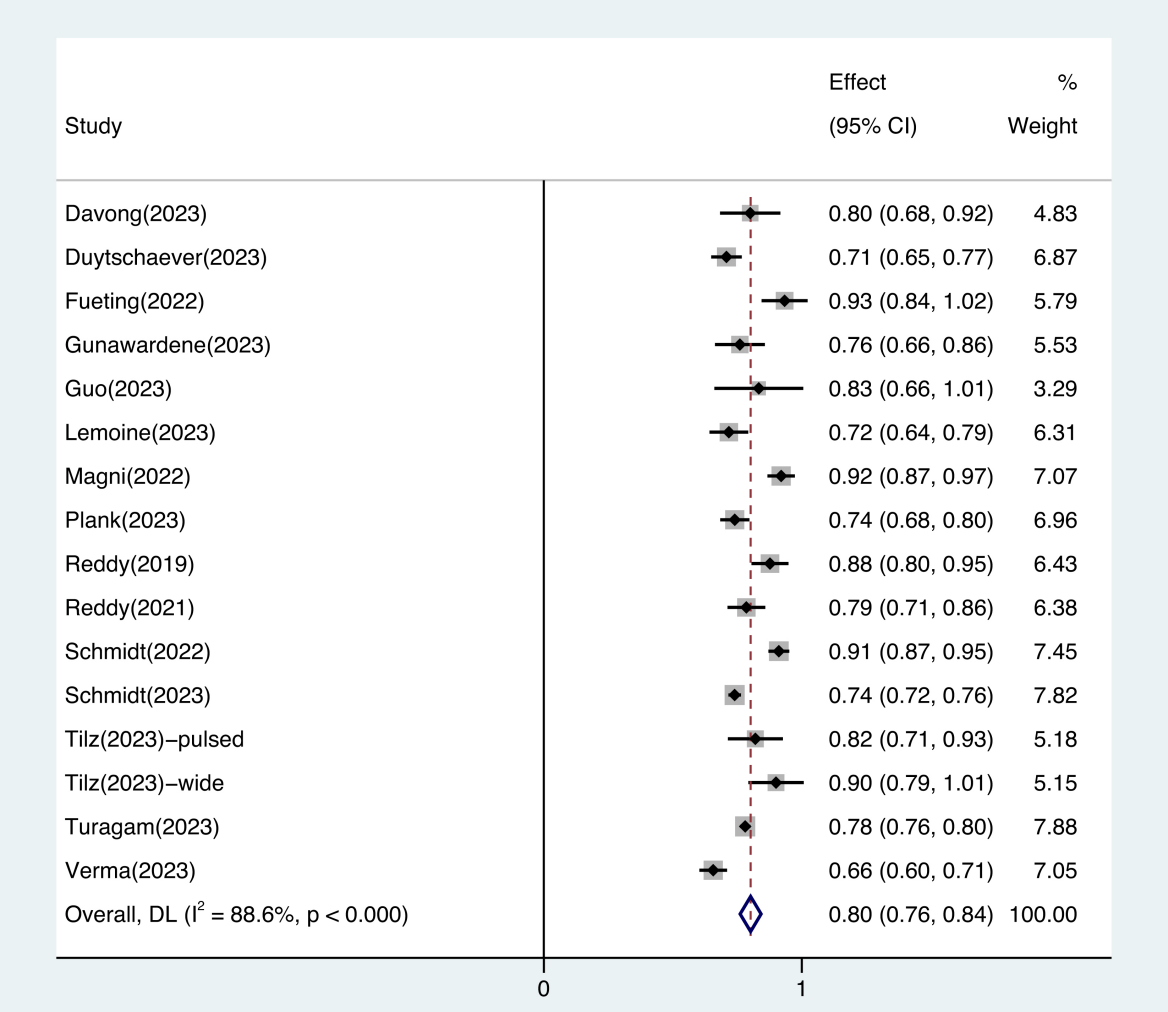
**The pooled free of arrhythmia recurrence rate of pulsed field ablation for atrial fibrillation patients**. The atrial arrhythmia recurrence-free rate was 0.80 (95% CI, 0.76–0.84), and heterogeneity was found between the studies (I^2^ = 88.6%). Abbreviation: CI, confidence interval; DL, DerSimonian and Laird approach.

The pooled estimates of free of arrhythmia recurrence rate of PFA are shown in Fig. 2. The atrial arrhythmia recurrence-free rate was 0.80 (95% CI, 0.76–0.84; Fig. 2), and heterogeneity was found between the studies (I^2^ = 88.6%, **Supplementary Fig. 2**). In patients with paroxysmal AF, the atrial arrhythmia recurrence-free rate was 0.80 (95% CI, 0.77–0.84, I^2^ = 76.7%; **Supplementary Fig. 3**); in patients with persistent AF, it was 0.68 (95% CI, 0.64–0.73, I^2^ = 61.4%; **Supplementary Fig. 4**). In studies with a follow-up period of at least 12 months, the atrial arrhythmia recurrence-free rate was 0.76 (95% CI, 0.72–0.79, I^2^ = 78.8%; **Supplementary Fig. 5**). The pooled complication rates were 0.03 (95% CI, 0.02–0.05; **Supplementary Fig. 6**). There was heterogeneity (I^2^ = 78.0%) in complications (**Supplementary Fig. 7**).

### 3.3 Effectiveness of PFA vs. Thermal Ablation

**Fig. 3.**
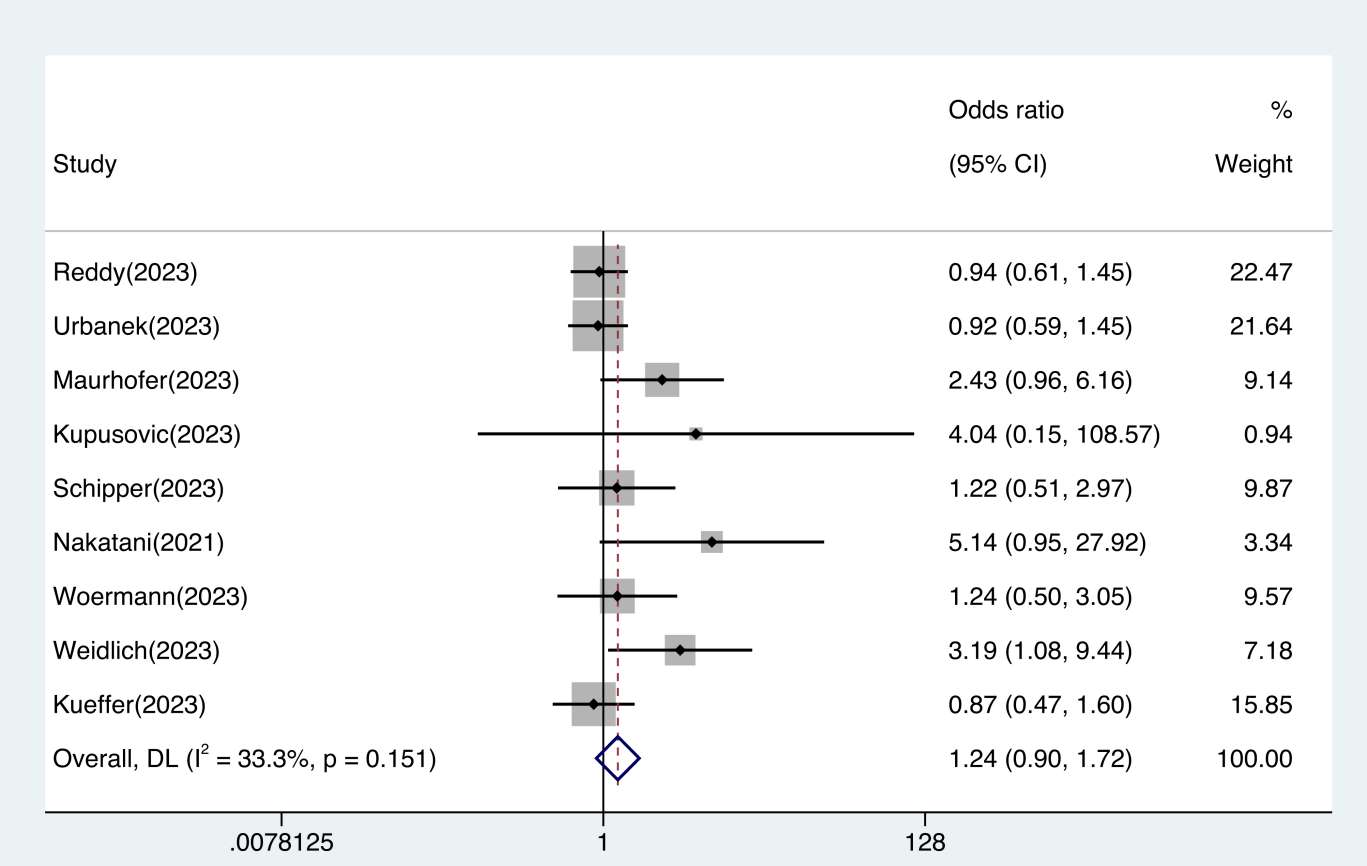
**Efficacy outcomes in patients treated with pulsed field ablation vs. thermal ablation**. The difference in efficacy between pulsed field ablation and traditional thermal ablation was not statistically significant (OR 1.24, 95% CI 0.90–1.72, I^2^: 33.3%). Abbreviation: CI, confidence interval; DL, DerSimonian and Laird approach; OR, odds ratio.

There was no significant difference in efficacy between the PFA and thermal ablation groups (OR 1.24, 95% CI 0.90–1.72, I^2^: 33.3%, Fig. 3). The publication bias evaluation revealed asymmetry in the funnel plot (**Supplementary Fig. 8**).

### 3.4 Safety of PFA vs. Thermal Ablation

**Fig. 4.**
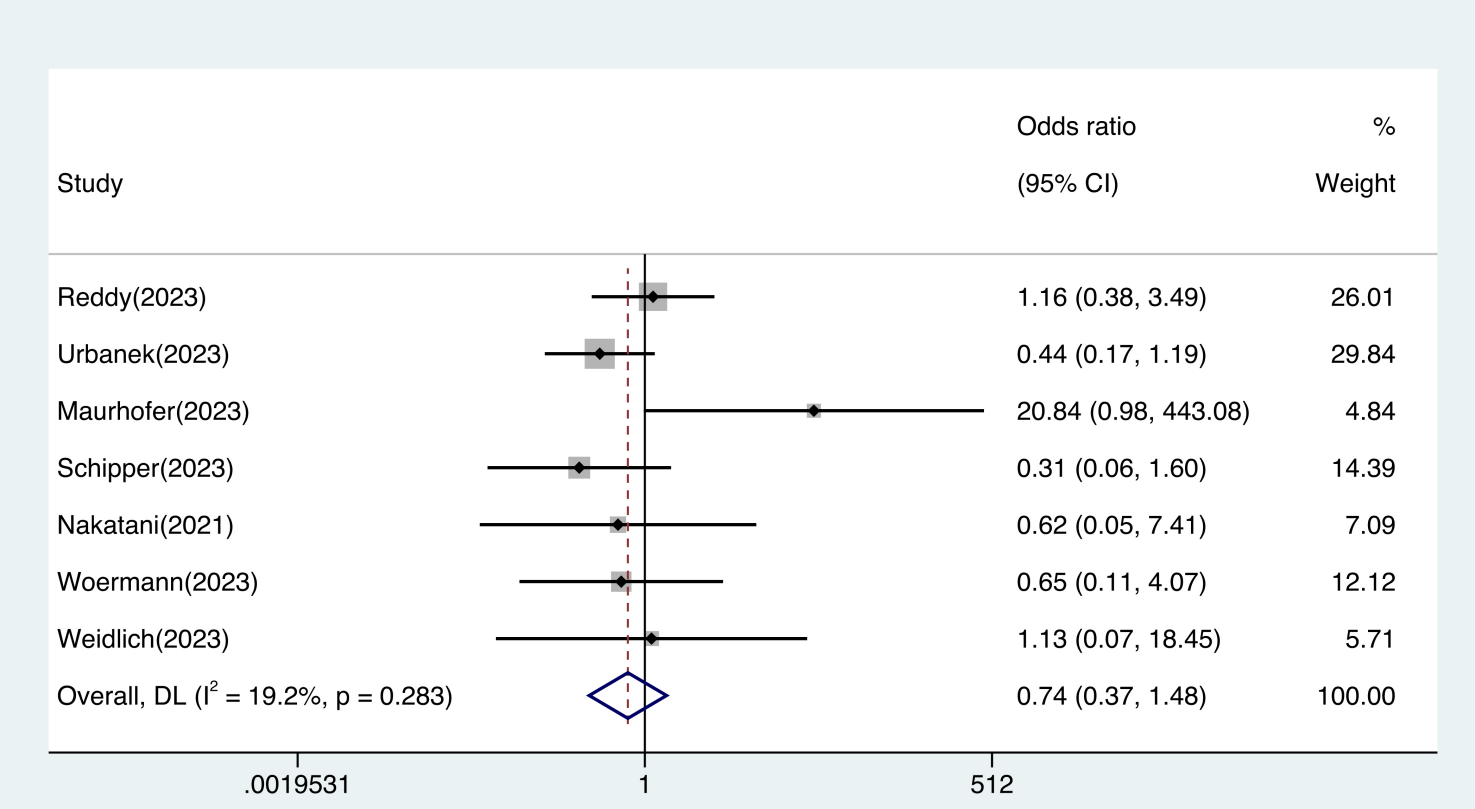
**Safety outcomes in patients treated with pulsed field ablation vs. thermal ablation**. The difference in safety between pulsed field ablation and traditional thermal ablation was not statistically significant (OR 0.74, 95% CI 0.37–1.48, I^2^: 19.2%). Abbreviation: CI, confidence interval; DL, DerSimonian and Laird approach; OR, odds ratio.

As shown in Fig. 4 and **Supplementary Fig. 9**, there was no significant safety difference between the PFA and thermal ablation groups (OR 0.74, 95% CI 0.37–1.48, I^2^: 19.2%) in complications. The publication bias assessment for this analysis revealed a significant asymmetry in the funnel plot.

### 3.5 Subgroup Analysis

As Table 3 shows, subgroup analysis showed that there was no significant efficacy difference between thermal ablation and PFA in paroxysmal AF (OR 1.46, 95% CI 0.83–2.56, I^2^: 52.2%, **Supplementary Fig. 10A**) and persistent AF (OR 1.02, 95% CI 0.64–1.61, I^2^: 0%, **Supplementary Fig. 10B**). In the mixed-type AF subgroup (paroxysmal, persistent), there was still no significant difference in their effectiveness (OR 1.61, 95% CI 0.94–2.75, I^2^: 0%, **Supplementary Fig. 10C**). In the safety analysis, there was no significant difference between thermal ablation and PFA in paroxysmal AF (OR 1.74, 95% CI 0.35–8.56, I^2^: 43.3%, **Supplementary Fig. 11A**), and only one article considered persistent AF alone. In the mixed-type AF subgroup, PFA had a slightly lower complication risk than thermal ablation (OR 0.47, 95% CI 0.22–0.99, I^2^: 0%, **Supplementary Fig. 11B**).

**Table 3.** **Comparison of primary efficacy and safety of thermal ablation and PFA in different subgroups**.

Factors			OR (95% CI)	I^2^	*p*
Type of AF					
	Paroxysmal AF				
		Efficacy	1.46 (0.83–2.56)	52.2%	0.099
		Safety	1.74 (0.35–8.56)	43.3%	0.171
	Persistent AF				
		Efficacy	1.02 (0.64–1.61)	0	0.444
	Mixed-type AF				
		Efficacy	1.61 (0.94–2.75)	0	0.472
		Safety	0.47 (0.22–0.99)	0	0.857
Follow-up time					
	<twelve month				
		Efficacy	2.19 (1.14–4.20)	3.6%	0.375
		Safety	0.72 (0.20–2.67)	0	0.940
	≥twelve month				
		Efficacy	1.01 (0.78–1.31)	2.1%	0.394
		Safety	0.84 (0.27–2.61)	58.9%	0.063
Ablation energy					
	Cryoballoon				
		Efficacy	1.12 (0.67–1.86)	44.2%	0.127
		Safety	0.70 (0.27–1.80)	31.6%	0.211
	Radiofrequency				
		Efficacy	1.64 (1.04–2.58)	0	0.499
		Safety	1.08 (0.45–2.58)	0	0.652

Abbreviation: AF, atrial fibrillation; OR, odds ratio; CI, confidence interval; PFA, pulsed field ablation.

**Fig. 5.**
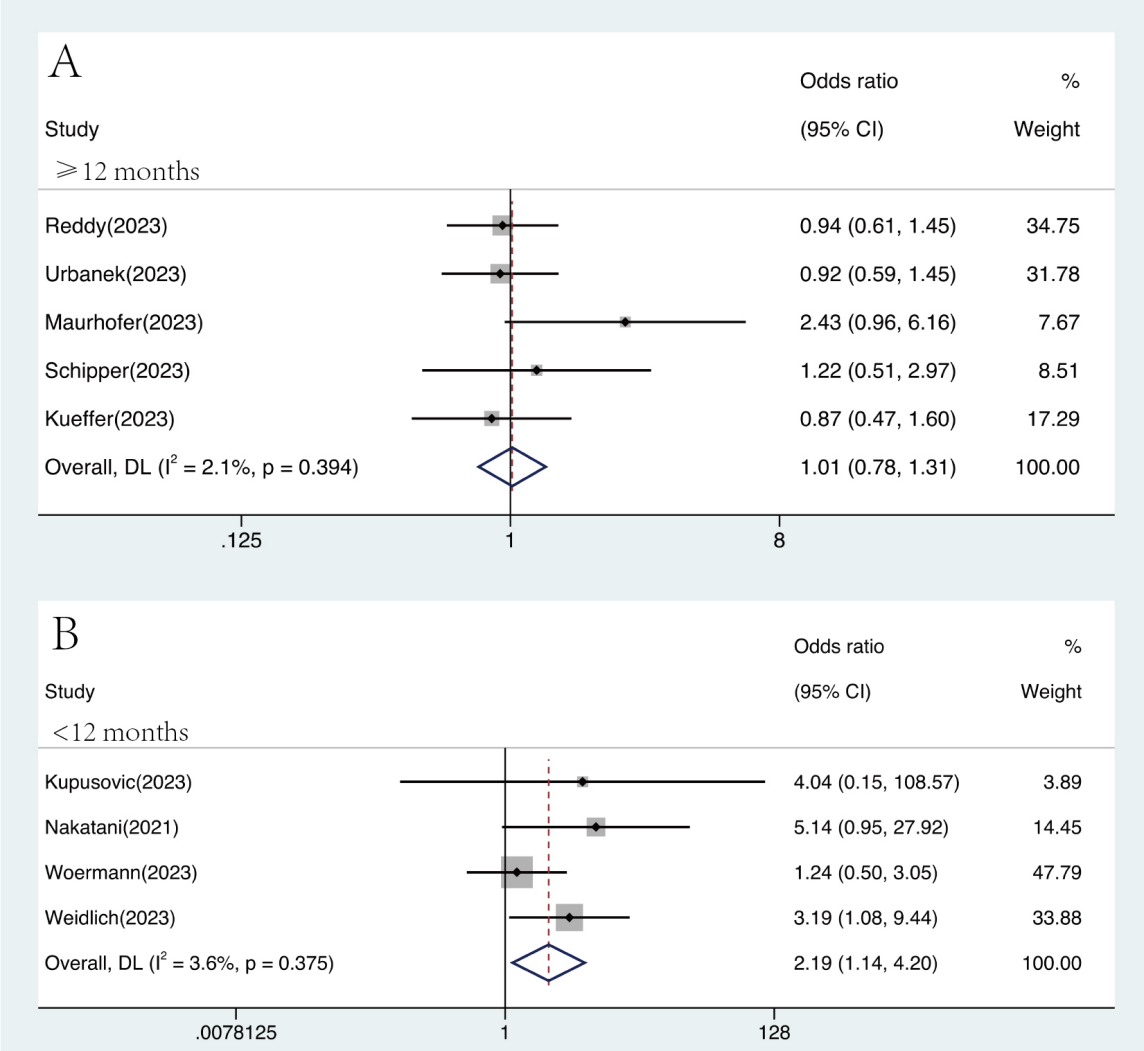
**Forest plots demonstrating the efficacy endpoint of atrial fibrillation patients for different follow-up durations**. In the subgroup with a follow-up period of less than 12 months, the efficacy of pulsed field ablation was superior to that of traditional thermal ablation (OR 2.19, 95% CI 1.14–4.20, I^2^ = 3.6%). Abbreviation: CI, confidence interval, DL, DerSimonian and Laird approach; OR, odds ratio.

Considering the different follow-up periods, we divided the studies into follow-up subgroups of <12 months and a follow-up subgroup of ≥12 months. In subgroup with ≥12 months of follow-up, there was no significant difference in the effectiveness (OR 1.01, 95% CI 0.78–1.31, Fig. 5A) and safety (OR 0.84, 95% CI 0.27–2.61, **Supplementary Fig. 12A**) of PFA compared with thermal ablation, with no heterogeneity (I^2^ = 2.1%) in the efficacy analysis and significant heterogeneity (I^2^ = 58.9%) in the safety analysis. Compared to thermal ablation, PFA had a better efficacy (OR 2.19, 95% CI 1.14–4.20, I^2^ = 3.6%, Fig. 5B) and similar safety (OR 0.72, 95% CI 0.20–2.67, I^2^ = 0%, **Supplementary Fig. 12B**) in the follow-up group of <12 months.

The samples were divided into radiofrequency ablation (RFA) and cryoballoon ablation (CBA) subgroups according to the thermal ablation method. PFA’s efficacy is similar to CBA (OR 1.12, 95% CI 0.67–1.86, I^2^: 44.2%, **Supplementary Fig. 13A**) but slightly superior to RFA (OR 1.64, 95% CI 1.04–2.58, I^2^: 0%, **Supplementary Fig. 13B**). No significant differences were observed between groups in the safety of PFA compared to CBA (OR 0.70, 95% CI 0.27–1.80, I^2^: 31.6%, **Supplementary Fig. 14A**) or RFA (OR 1.08, 95% CI 0.45–2.58, I^2^: 0%, **Supplementary Fig. 14B**).

## 4. Discussion

PFA, a novel application of irreversible electroporation technology, preferentially ablates myocardial tissue, thereby protecting the adjacent tissues [9, 35, 36], and is considered to be an ablation technique with theoretical advantages. In this study, we found that PFA had a low recurrence rate of atrial arrhythmia after AF ablation while satisfying procedural safety and is not inferior to traditional thermal ablation both in terms of efficacy or safety. The PFA group achieved higher rates of atrial arrhythmia-freedom in the follow-up of less than 12 months. In the study on mixed-type AF, PFA appeared to be safer than thermal ablation. Additionally, the efficacy of PFA is slightly better than that of RFA.

Thermal ablation has been used in the clinical treatment of AF for approximately 30 years. Compared with antiarrhythmic drugs, thermal ablation not only improves the quality of life of patients but also significantly improves their prognosis [2]. Although thermal ablation is already very safe, it can cause damage to the esophagus, phrenic nerve, and pulmonary veins; occasionally, these lesions may be life-threatening [37].

The incidence of complications caused by traditional thermal ablation is approximately 6.29%, and the in-hospital mortality rate is approximately 0.49% [4]. This is a fairly good value; however, PFA can further improve the safety of the ablation procedure in theory, particularly by reducing the incidence of serious complications arising from damage to adjacent tissues. In terms of safety, Grosse *et al*. [35] found through esophageal endoscopy that PFA hardly causes esophageal and periesophageal injury. Pansera *et al*. [38] found that phrenic nerve dysfunction after PFA is mostly transient and can recover spontaneously. With appropriate parameter settings, PFA can effectively avoid phrenic nerve injury [36]. Moreover, compared with thermal ablation, PFA reduces the degree of pulmonary vein constriction [39] and the risk of pulmonary vein stenosis [40]. In a small sample study by Reinsch *et al*. [41], following PFA, only 3% of patients showed fewer cerebellar micro lesions, compared with 15.8% following thermal ablation [42]. Therefore, whether PFA positively impacts long-term cognitive decline after ablation could be a follow-up research direction.

However, the frequency of cardiac tamponade was slightly higher in the PFA group compared with the thermal ablation group [14, 21]. In the study of Reddy *et al*. [21], there was a case of death caused by pericardial tamponade due to a lack of experience. The two cases of pericardial tamponade that appeared in the study by Maurhofer *et al*. [15] were not related to energy delivery. After changing their workflow, no further pericardial tamponade exited. Additionally, the X-ray exposure time for PFA was slightly longer than CBA and significantly longer than RFA [15, 21]. This is attributed to the need for X-ray guidance to locate the pulmonary veins, the immaturity of the system and workflow, and the operators’ unfamiliarity with the PFA procedure.

Zhang *et al*. [43] suggested that coronary spasms, one of the common complications of PFA, are mostly associated with mitral isthmus ablation. In a study by Turagam *et al*. [11], in addition to one case of coronary artery spasm caused by mitral valve isthmus ablation, another case of remote coronary artery spasm was found, which was believed to be driven by the autonomic nervous system. The current research findings suggest that coronary spasms caused by PFA rarely lead to serious adverse outcomes and mostly improve after the administration of nitroglycerin.

Our research results indicate that the complications of PFA are approximately 3%, which is slightly less than the 6.29% reported for thermal ablation [4]. However, no such differences have been found in comparative studies. Through subgroup analysis, we noticed no significant difference in the risk of complications between PFA and RFA or CBA, and the difference in complications was not significant when considering paroxysmal AF alone. Studies that include both paroxysmal and persistent AF have shown that PFA has slightly higher safety. But this result may not be reliable because of the small number of studies included and the insufficient sample size. In our study, neither the PFA group nor the thermal ablation group experienced severe procedural complications such as atrial esophageal fistula and pulmonary vein stenosis. However, more persistent paralysis of the phrenic nerve was observed in the thermal ablation group, especially in the CBA group, but not in the PFA group, indicating a protective effect of PFA on the phrenic nerve [5, 14, 21]. In addition, there were no significant differences in other perioperative complications, such as stroke, transient ischemic attack, myocardial infarction, and vascular pathway complications [21].

The overall incidence of complications was similar in the PFA and thermal ablation groups. This may be because thermal ablation routinely combines auxiliary ablation strategies such as esophageal temperature monitoring, and with the development of ablation navigation systems, equipment, and technology, the probability of complications is relatively low [10]. Second, there is currently no consensus or established standard for the PFA procedure, and operators lack sufficient experience. With the increase in the number of PFA procedures and publications of research, this issue can be resolved. Finally, the sample size of the current study may have been insufficient to compare the incidence of complications.

At present, the efficacy of a single thermal ablation for paroxysmal AF is approximately 70% [44], whereas that for persistent or long-term persistent AF is only 43% [6]. Previous researchers have made many attempts to improve the efficacy of catheter ablation in patients with AF. For example, anatomically guided ablation is performed based on specific histological origins [45], and precise ablation is performed based on imaging or voltage mapping to find targets [46]; however, most of these do not improve procedure efficacy. PFA has excellent acute procedural efficacy, with an acute pulmonary vein isolation (PVI) rate of over 99% [11, 12] and a single-shot isolation rate of over 90% [33].

Our meta-analysis of only PFA articles showed that the effectiveness of PFA in AF was approximately 80%. In comparative studies, there was no significant difference in the efficacy of PFA compared with thermal ablation. However, PFA performed better than thermal ablation in studies with a follow-up period of <12 months. This may be related to the milder tissue inflammation caused by PFA in the blanking period, leading to a reduction in recurrence [18]. It may also suggest that PFA cannot form a durable PVI lesion under the current procedural setting. Nakatani *et al*. [17] found that PVI generated by PFA rarely experienced reconnection at 3 months, which may suggest that lesions formed by PFA may rapidly invalidate after 3 months, resulting in an efficacy similar to that of thermal ablation. Additionally, our subgroup analysis revealed that the efficacy of PFA is slightly higher than that of RFA. This result could be attributed to the fact that most studies in this subgroup had small sample sizes and short follow-up periods, which have influenced the conclusions.

Although current studies have not shown significant improvements in efficacy and safety compared with thermal ablation, PFA still has some advantages that must be further revealed. PFA significantly shortened total procedural duration, and compared to CBA, it reduced fluoroscopy agent dosage. Compared with high-power short-duration RFA, PFA still has a shorter procedure time [16, 19]. The above can improve patient tolerance to the procedure. Additionally, for operators, PFA has a brief learning curve [5], which means that the repeatability of the operation is higher, which is conducive to its promotion and application. Therefore, PFA can reduce the difficulty of the procedure, making it easier for both doctors and patients to accept it, and enhance the feasibility of its promotion in grassroots hospitals.

Furthermore, flexible and additional ablation strategies are needed to balance the occurrence of complications caused by an excessive ablation area, high ablation power, and prolonged ablation time [46]. Owing to the compromise between efficacy and safety, thermal ablation methods often do not adopt more aggressive settings. PFA has the advantages of both CBA and RFA, allowing for more flexible additional ablation while ensuring a shorter procedure time. To form more durable lesions, PFA can perform more rigorous ablation settings while ensuring safety and disrupting the balance between efficacy and safety. In an unpublished study by Adelino *et al*. [47], additional ablation with PFA significantly increased the proportion of recovery of sinus rhythm, reduced the cardioversion rate during the procedure, and had half the complications compared with thermal ablation. PFA not only performs well in PVI but also demonstrates appreciable efficacy in additional atrial tissue ablation, such as the left atrial posterior wall [48] and mitral isthmus [26]. However, rigorous settings must consider the potential for severe acute kidney injury due to hemolysis that could arise from excessive application of PFA [49].

Additionally, PFA preferentially kills myocardial cells and retains the original structure of the extracellular matrix, resulting in a reduced postoperative inflammatory response, fewer activated fibroblasts, and less chronic left atrial fibrosis [17, 18]. After 3 months of the procedure, the decrease in strain in the left atrial posterior wall and pulmonary vein antra caused by PFA will recover, but thermal ablation will not [17]. This suggests that PFA is advantageous for improving the long-term prognosis of patients with heart failure.

The present study had a few limitations. First, only one eligible study was an RCT; the others were cohort studies. Second, only some of the included studies performed esophageal endoscopy or magnetic resonance imaging, for which the examination was insufficient; thus, some of the complications may not have been discovered, resulting in a low incidence of complications. Third, the study did not distinguish between specific ablation devices for the general review of PFA. Fourth, some outcomes in the eligible studies were not reported in detail and were not subdivided; thus, some subgroup analyses, including too few studies, and did not allow for more in-depth research, such as gender differences in efficacy or safety outcomes. Finally, some of the eligible studies for calculating our conclusions were unpublished, and there may be slight differences in the conclusions after the studies were published. For subsequent research, we need not only large-sample, high-quality, and long-term follow-up studies to confirm the safety and efficacy of PFA but also reliable studies comparing the safety and efficacy of the two ablation energies in additional ablation to provide more evidence.

## 5. Conclusions

PFA is an effective and safe ablation method for AF. Compared to traditional thermal ablation, PFA has similar efficacy and safety. Additionally, PFA helps to reduce procedure time, decrease the learning cost for operators, and still has potential advantages worth further exploration, with the expectation of becoming a common choice for the treatment of AF in clinical practice.

## Availability of Data and Materials

The data that support the findings of this study are available from the corresponding author upon reasonable request.

## Supplementary Material

Supplementary material associated with this article can be found, in the online version, at https://doi.org/10.31083/j.rcm2511415.
