# Supplementary material for: Safety and Efficacy of Pulse Field Ablation in the Treatment of Atrial Fibrillation and Its Comparison with Traditional Thermal Ablation: A Systematic Review and Meta-Analysis
Source: Rev Cardiovasc Med. 2024 Nov 21;25(11):415. doi: 10.31083/j.rcm2511415 (PMC11607503; doi:10.31083/j.rcm2511415)
Supplement: Supplementary file 1 [file 2153-8174-25-11-415-s1.zip › Supplementary figure(s).docx]

**Page 2: Supplementary Fig. 1:** Quality assessment for randomized controlled study.

**Page 3: Supplementary Fig. 2:** Funnel plots for the pooled free of arrhythmia recurrence rate of pulsed field ablation.

**Page 4: Supplementary Fig. 3:** The pooled free of arrhythmia recurrence rate of pulsed field ablation for paroxysmal atrial fibrillation patients. Abbreviation: CI = confidence interval, DL = DerSimonian and Laird approach.

**Page 5: Supplementary Fig. 4:** The pooled free of arrhythmia recurrence rate of pulsed field ablation for persistent atrial fibrillation patients. Abbreviation: CI = confidence interval, DL = DerSimonian and Laird approach.

**Page 6: Supplementary Fig. 5:** The pooled free of arrhythmia recurrence rate of pulsed field ablation for atrial fibrillation patients in studies with a follow-up period of at least 12 months. Abbreviation: CI = confidence interval, DL = DerSimonian and Laird approach.

**Page 7: Supplementary Fig. 6:** The pooled safety indexes of pulsed field ablation for atrial fibrillation patients. Abbreviation: CI = confidence interval

**Page 8: Supplementary Fig. 7:** Funnel plots for the pooled complication rate of pulsed field ablation.

**Page 9: Supplementary Fig. 8:** Funnel plots and sensitive analysis for the efficacy endpoint of pulsed field ablation vs. thermal ablation. Abbreviation: CI = confidence interval

**Page 10: Supplementary Fig. 9:** Funnel plots and sensitive analysis for the safety endpoint of pulsed field ablation vs. thermal ablation. Abbreviation: CI = confidence interval , DL = DerSimonian and Laird approach.

**Page 11: Supplementary Fig. 10:** Forest plots demonstrating the efficacy endpoint of atrial fibrillation patients for different type of atrial fibrillation. Abbreviation: PAF = paroxysmal atrial fibrillation; PSAF = persistent atrial fibrillation; Mixed-AF = mixed type atrial fibrillation; CI = confidence interval, DL = DerSimonian and Laird approach.

**Page 12: Supplementary Fig. 11:** Forest plots demonstrating the safety endpoint of atrial fibrillation patients for different type of atrial fibrillation. Abbreviation: PAF = paroxysmal atrial fibrillation; Mixed-AF = mixed type atrial fibrillation; CI = confidence interval, DL = DerSimonian and Laird approach.

**Page 13: Supplementary Fig. 12:** Forest plots demonstrating the safety endpoint of atrial fibrillation patients for different follow-up duration. Abbreviation: CI = confidence interval, DL = DerSimonian and Laird approach.

**Page 14: Supplementary Fig. 13:** Forest plots demonstrating the efficacy endpoint of atrial fibrillation patients for different ablation energy. Abbreviation: CBA = cryoballoon ablation; RFA = radiofrequency ablation; CI = confidence interval, DL = DerSimonian and Laird approach.

**Page 15: Supplementary Fig. 14:** Forest plots demonstrating the safety endpoint of atrial fibrillation patients for different ablation energy. Abbreviation: CBA = cryoballoon ablation; RFA = radiofrequency ablation; CI = confidence interval, DL = DerSimonian and Laird approach.

**Supplementary Fig. 1:** Quality assessment for randomized controlled study.
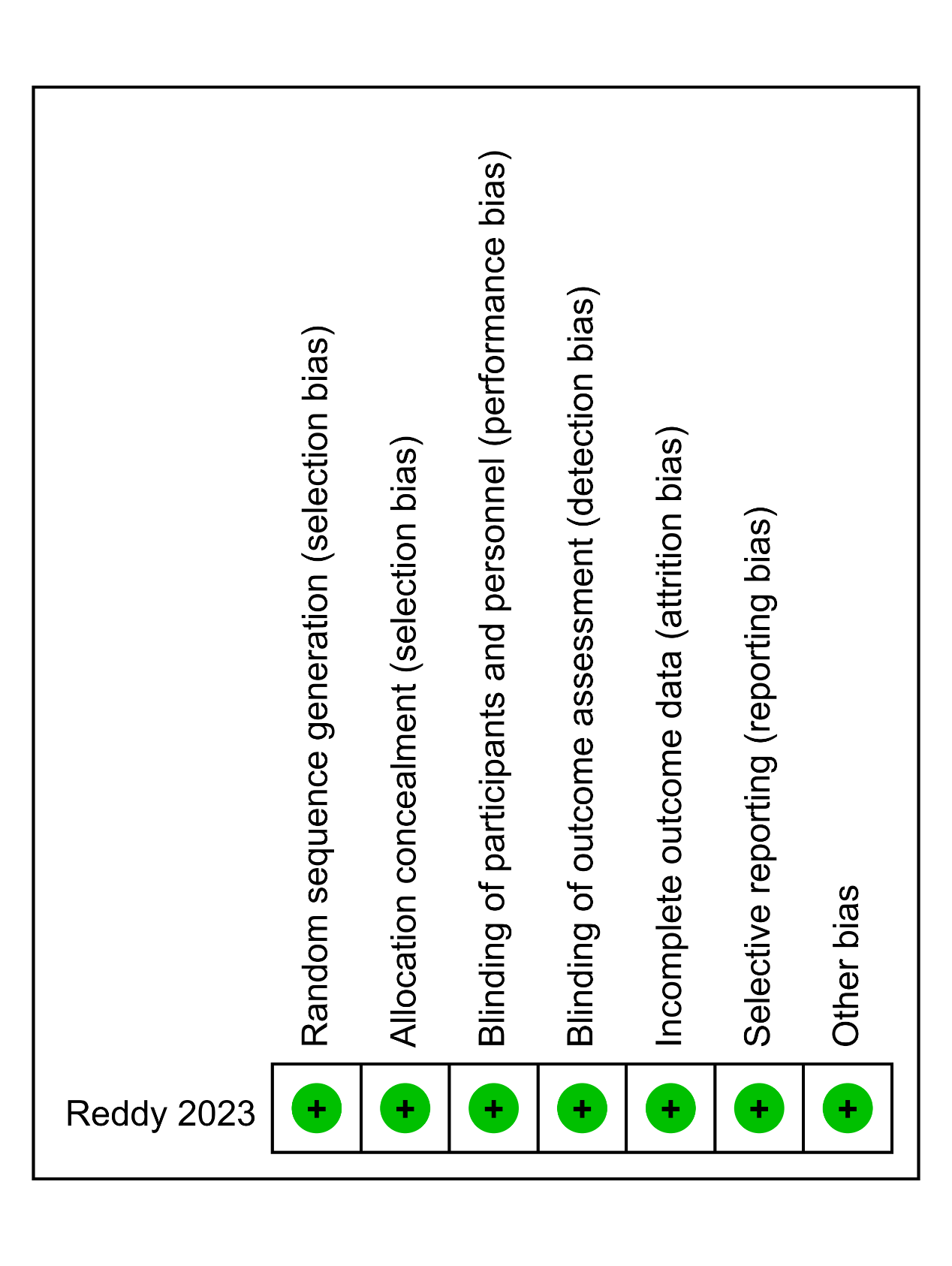


**Supplementary Fig. 2:** Funnel plots for the pooled free of arrhythmia recurrence rate of pulsed field ablation.

**
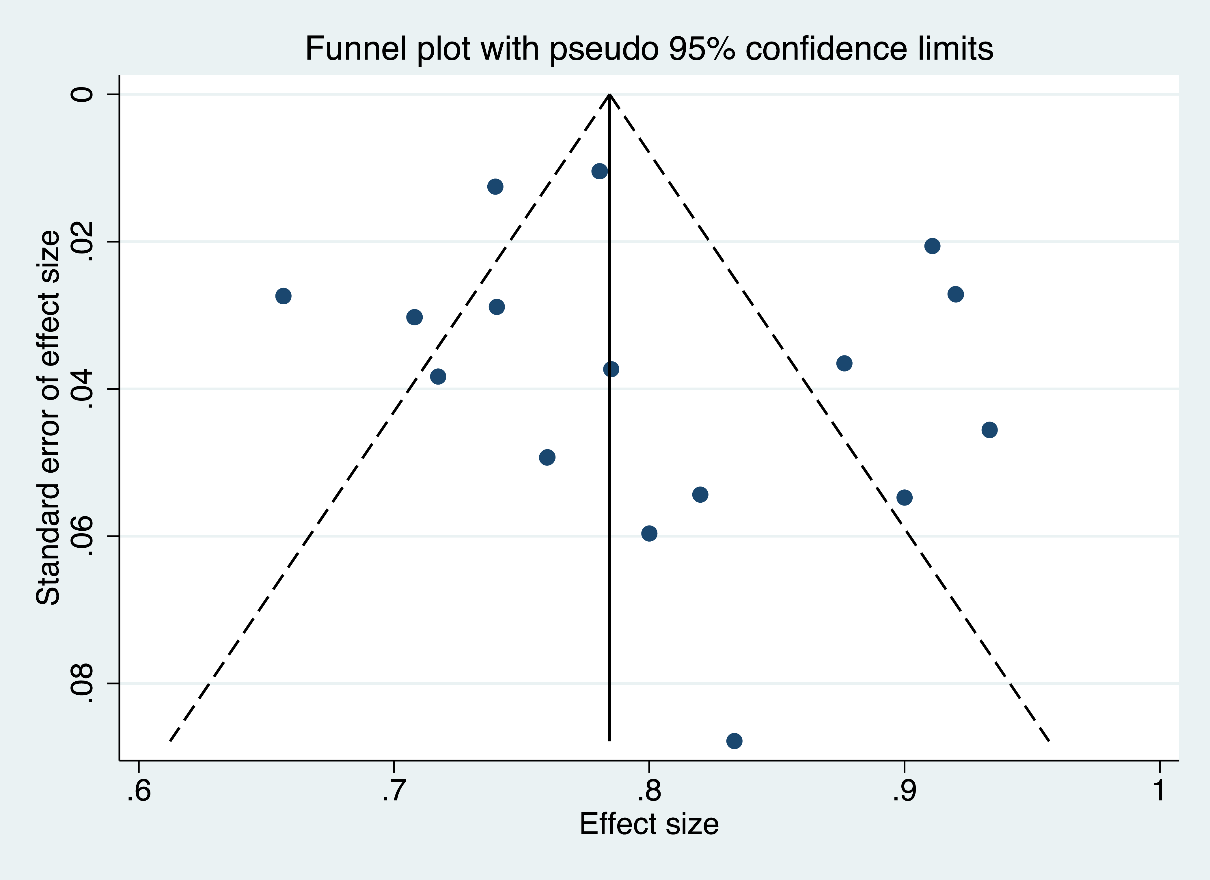
**

**Supplementary Fig. 3: The pooled free of arrhythmia recurrence rate of pulsed field ablation for paroxysmal atrial fibrillation patients.**
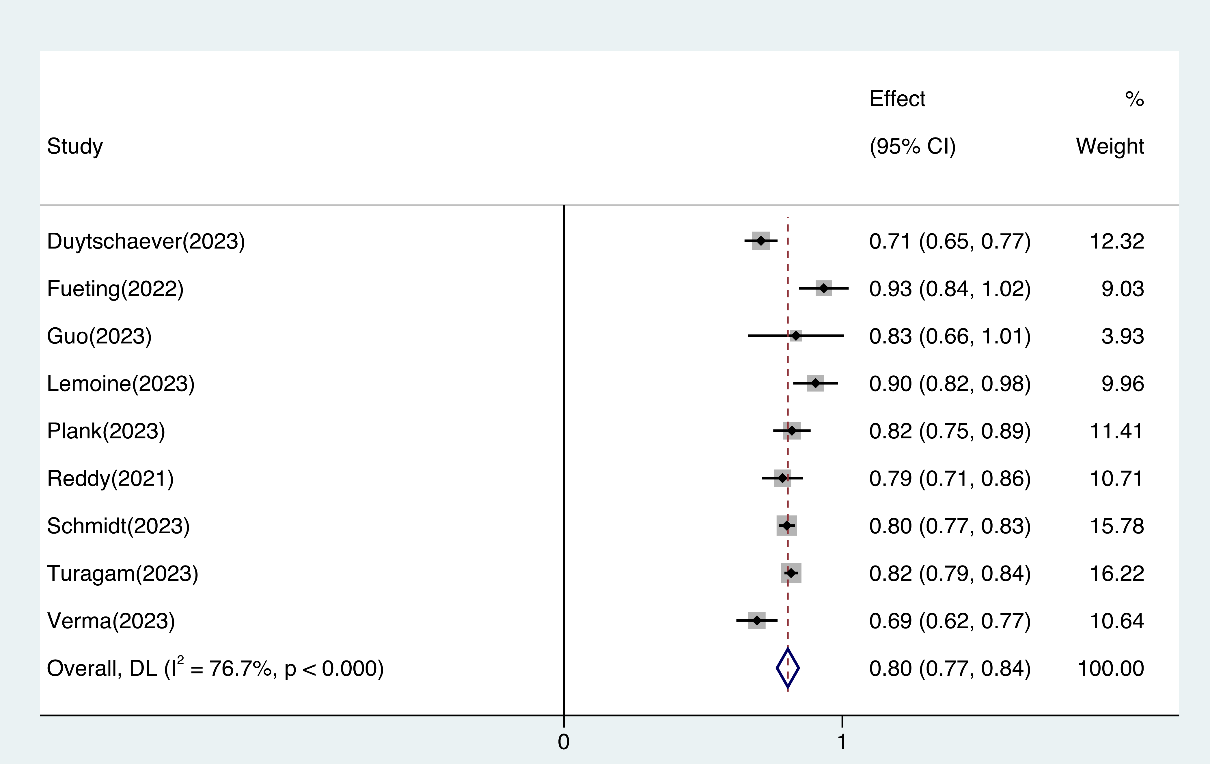


Abbreviation: CI = confidence interval, DL = DerSimonian and Laird approach.

**Supplementary Fig. 4: The pooled free of arrhythmia recurrence rate of pulsed field ablation for persistent atrial fibrillation patients.**
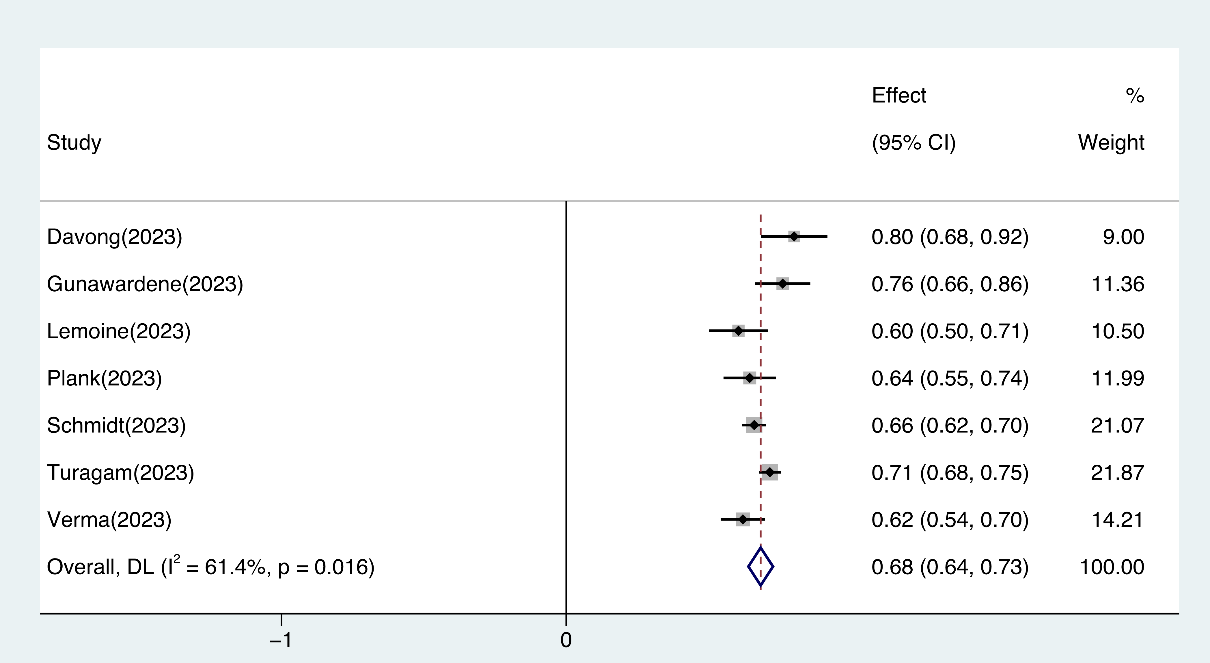


Abbreviation: CI = confidence interval, DL = DerSimonian and Laird approach.

**Supplementary Fig. 5: The pooled free of arrhythmia recurrence rate of pulsed field ablation for atrial fibrillation patients in studies with a follow-up period of at least 12 months.
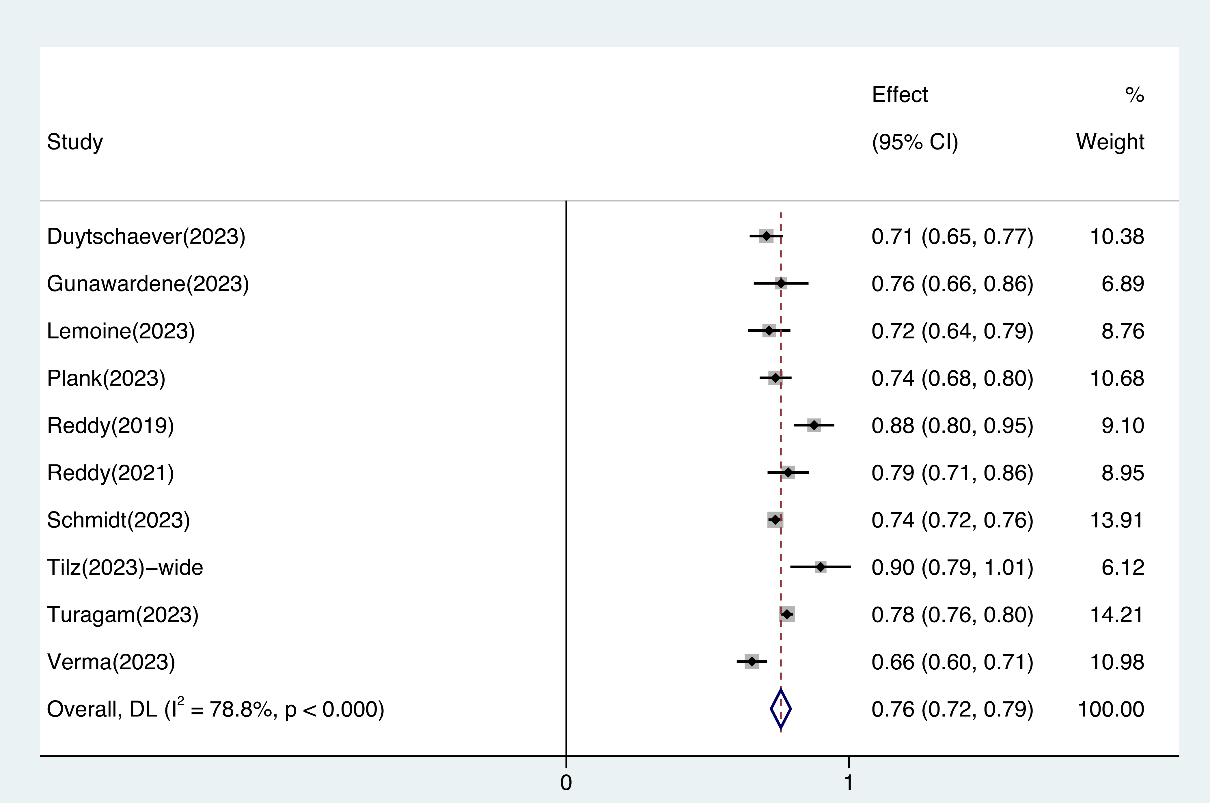
**

Abbreviation: CI = confidence interval, DL = DerSimonian and Laird approach.

**Supplementary Fig. 6:** The pooled safety indexes of pulsed field ablation for atrial fibrillation patients. **
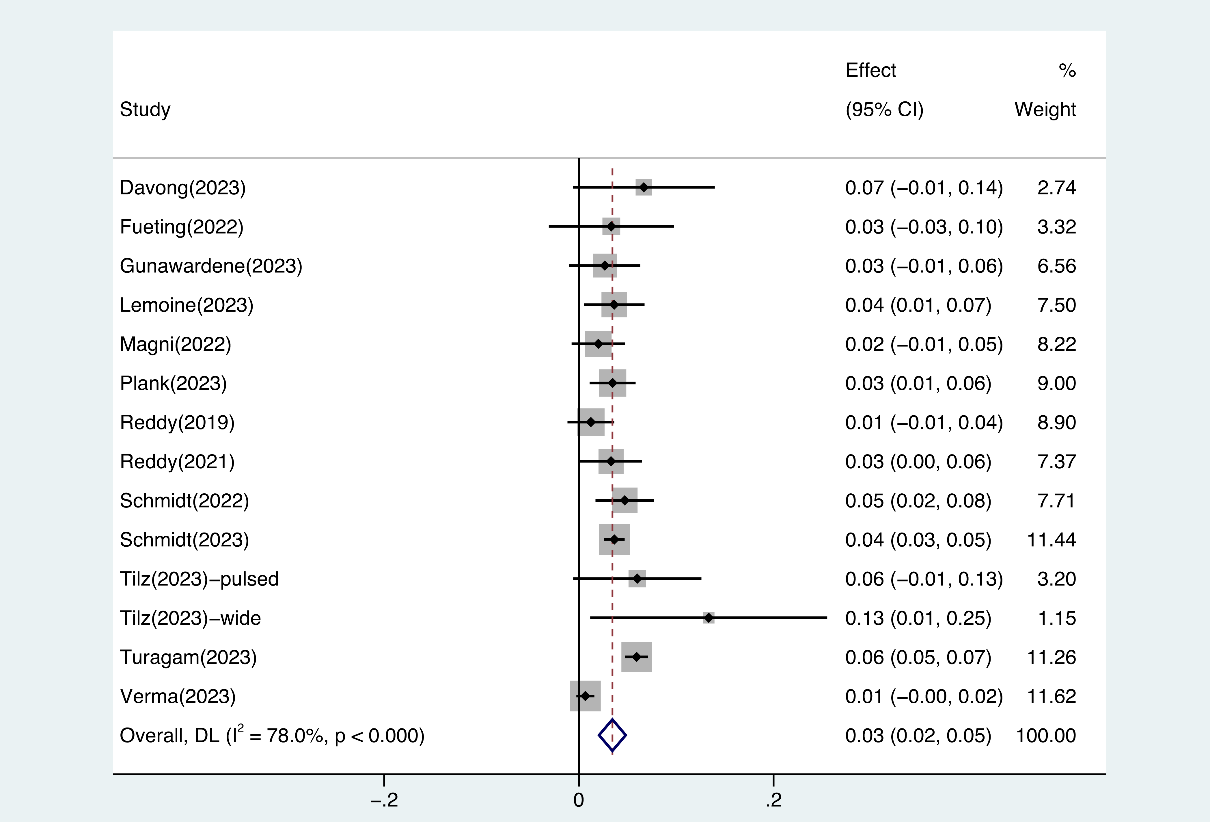
**

Abbreviation: CI = confidence interval, DL = DerSimonian and Laird approach.

**Supplementary Fig. 7:** Funnel plots for the pooled complication rate of pulsed field ablation.**
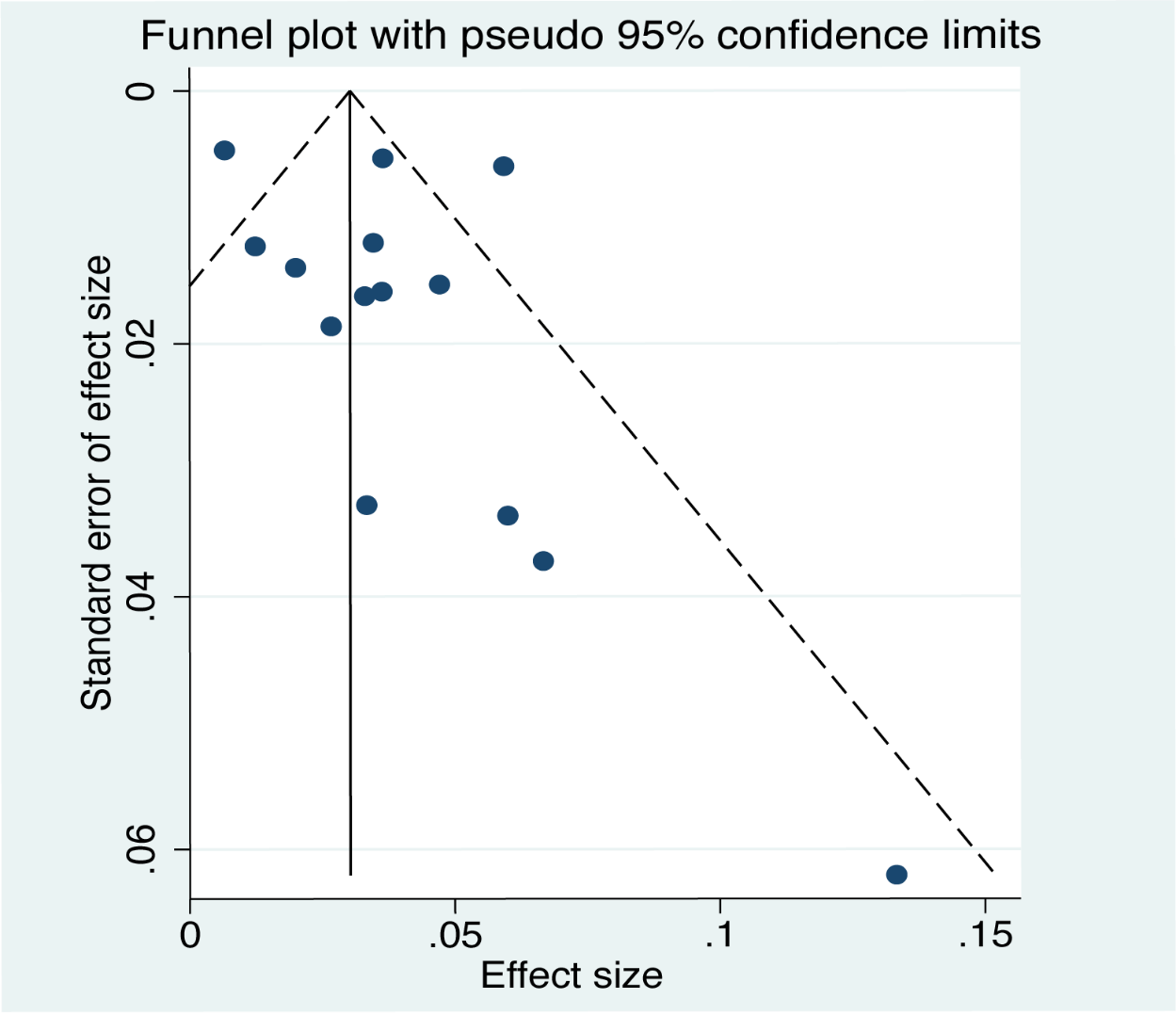
**

**Supplementary Fig. 8:** Funnel plots and sensitive analysis for the efficacy endpoint of pulsed field ablation vs. thermal ablation.
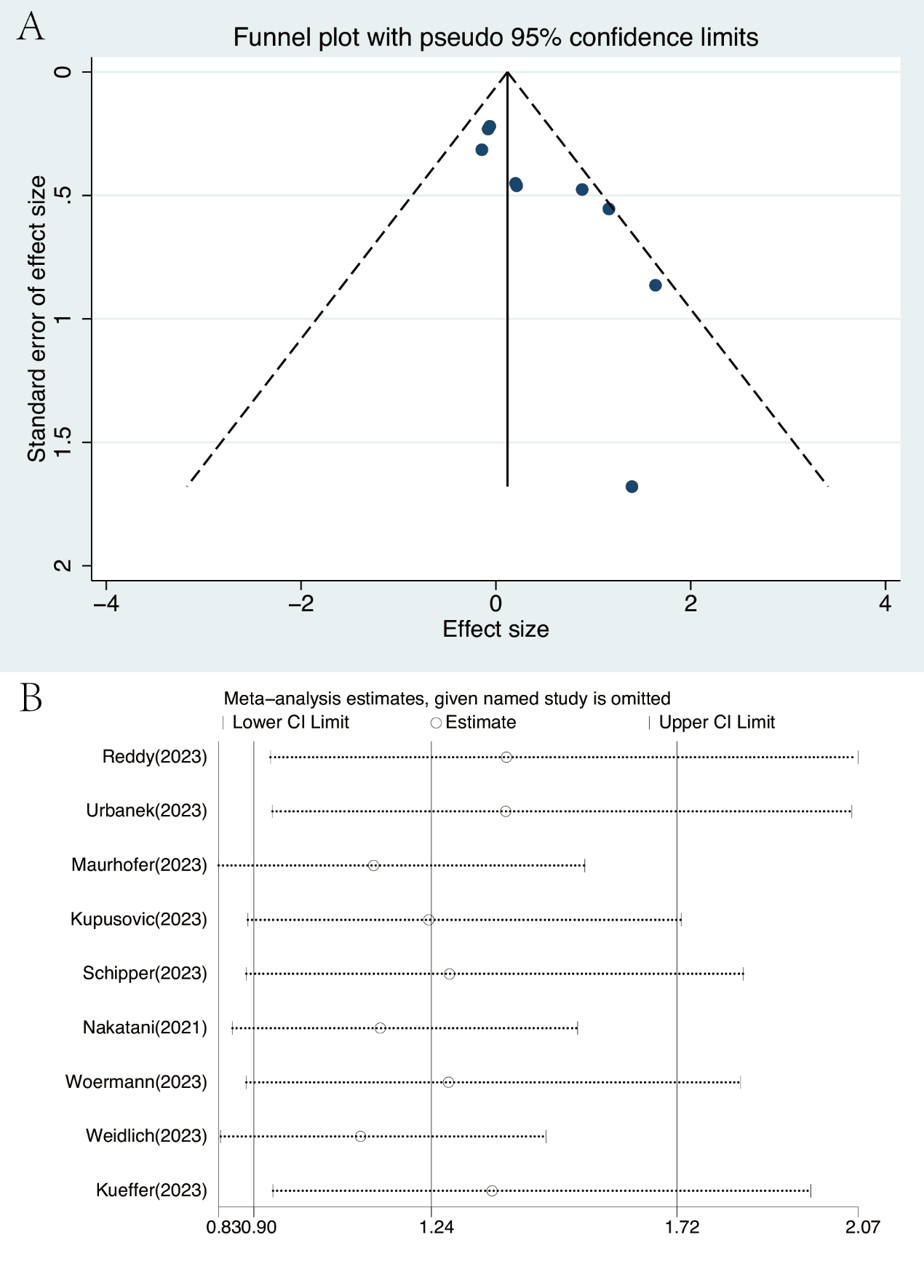


Abbreviation: CI = confidence interval

**Supplementary Fig. 9:** Funnel plots and sensitive analysis for the safety endpoint of pulsed field ablation vs. thermal ablation.
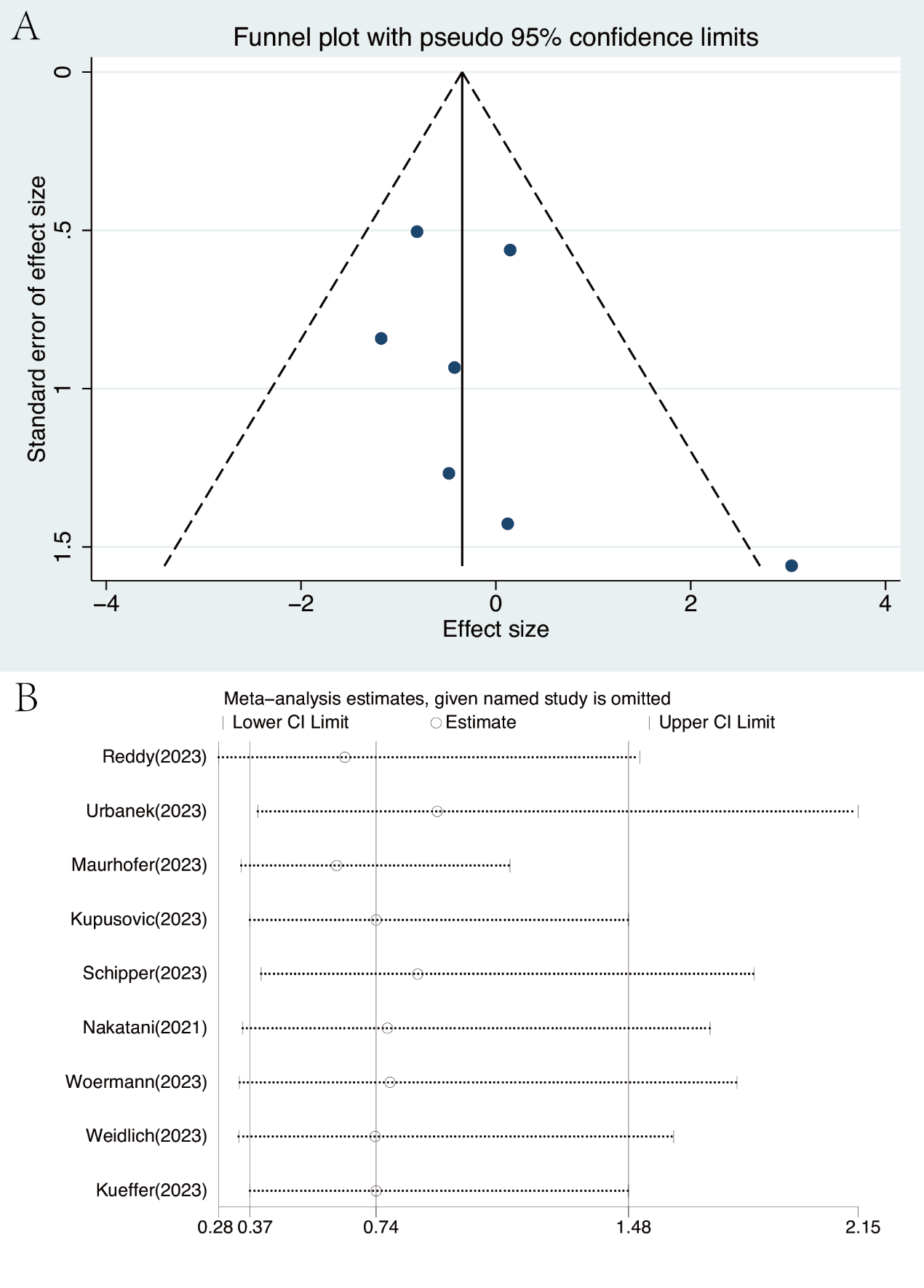


Abbreviation: CI = confidence interval

**Supplementary Fig. 10:** Forest plots demonstrating the efficacy endpoint of atrial fibrillation patients for different type of atrial fibrillation. **
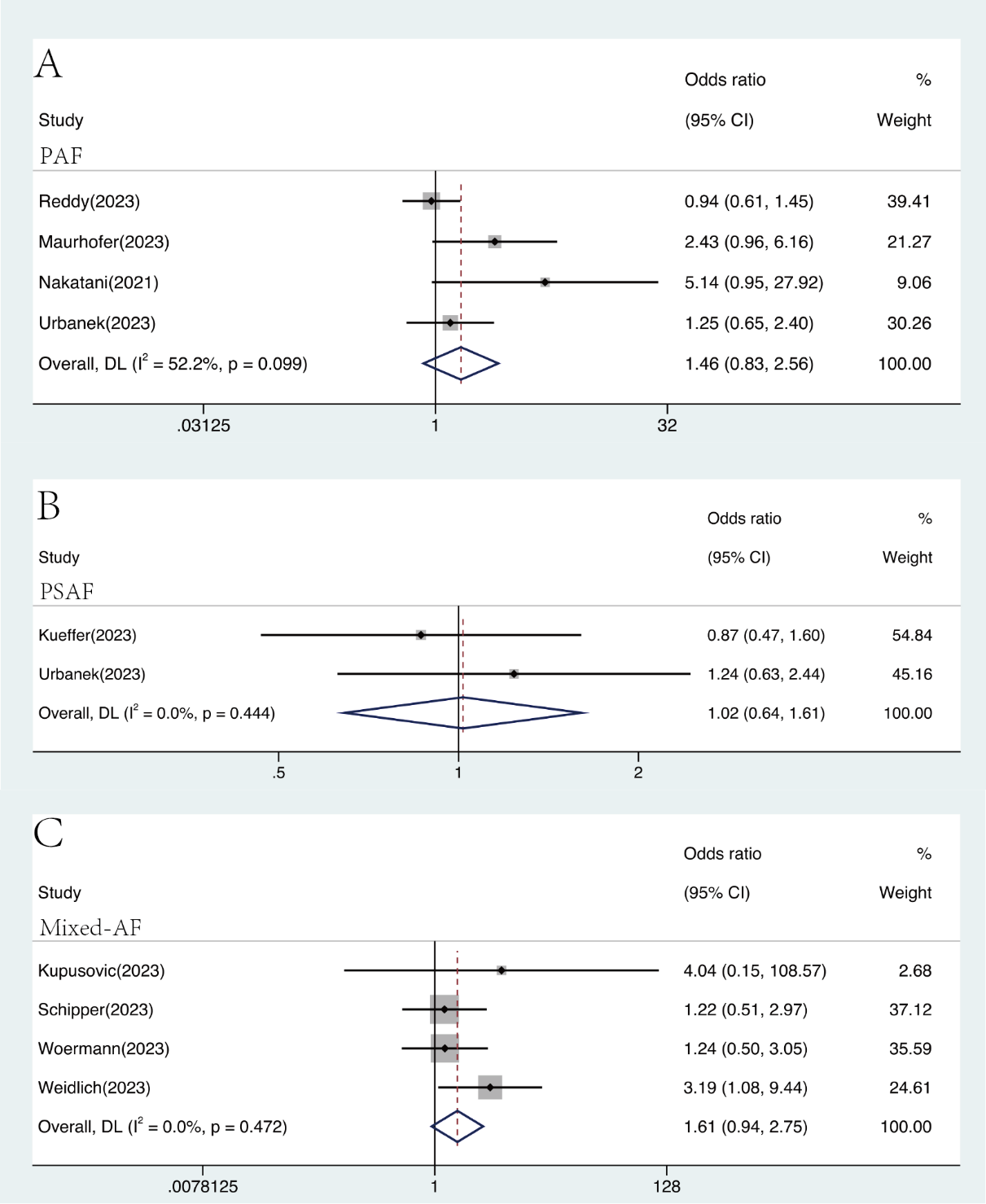
**

Abbreviation: PAF = paroxysmal atrial fibrillation; Mixed-AF = mixed type atrial fibrillation; CI = confidence interval, DL = DerSimonian and Laird approach.

**Supplementary Fig. 11:** Forest plots demonstrating the safety endpoint of atrial fibrillation patients for different type of atrial fibrillation.
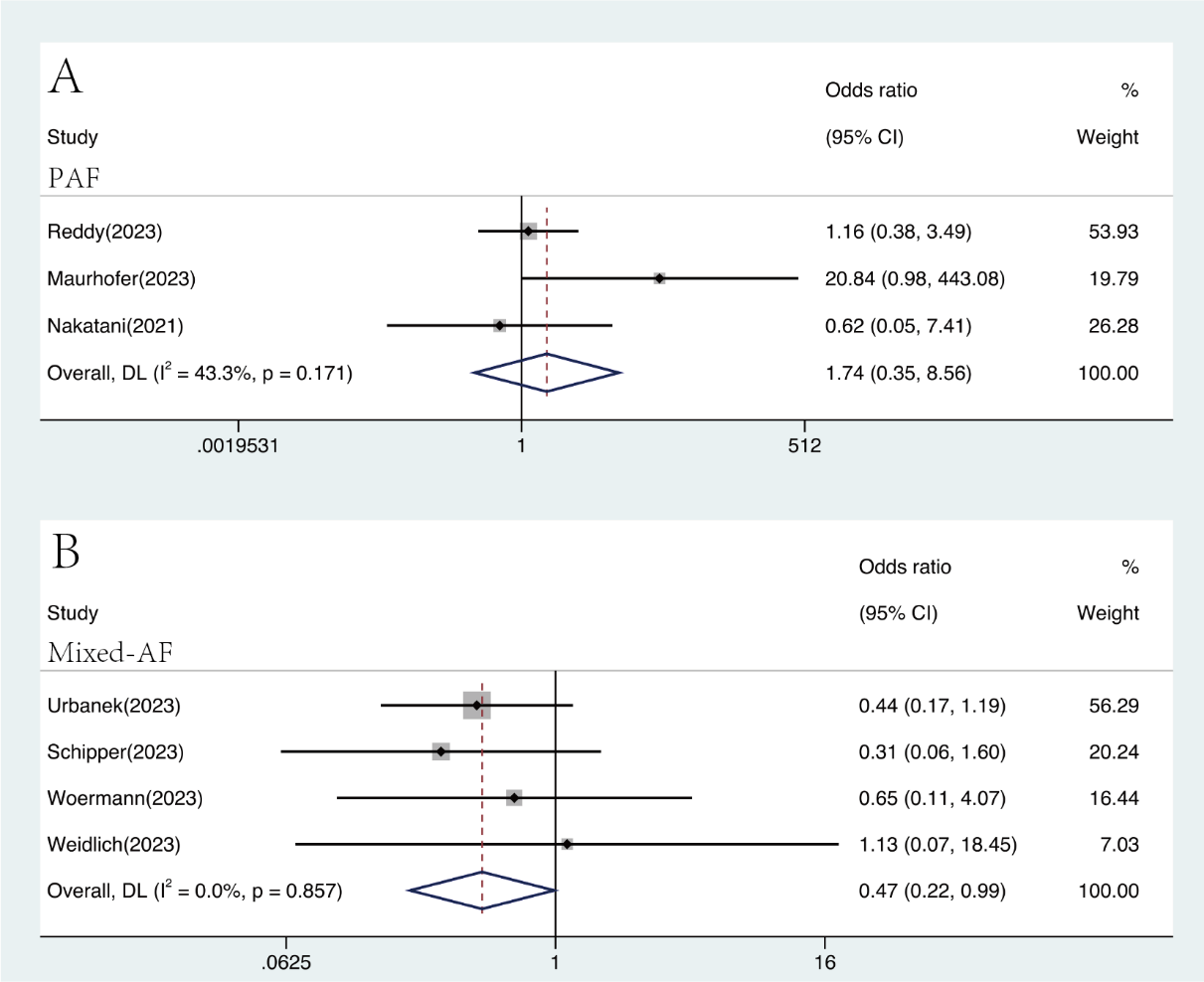


Abbreviation: PAF = paroxysmal atrial fibrillation; Mixed-AF = mixed type atrial fibrillation; CI = confidence interval, DL = DerSimonian and Laird approach.

**Supplementary Fig. 12:** Forest plots demonstrating the safety endpoint of atrial fibrillation patients for different follow-up duration.
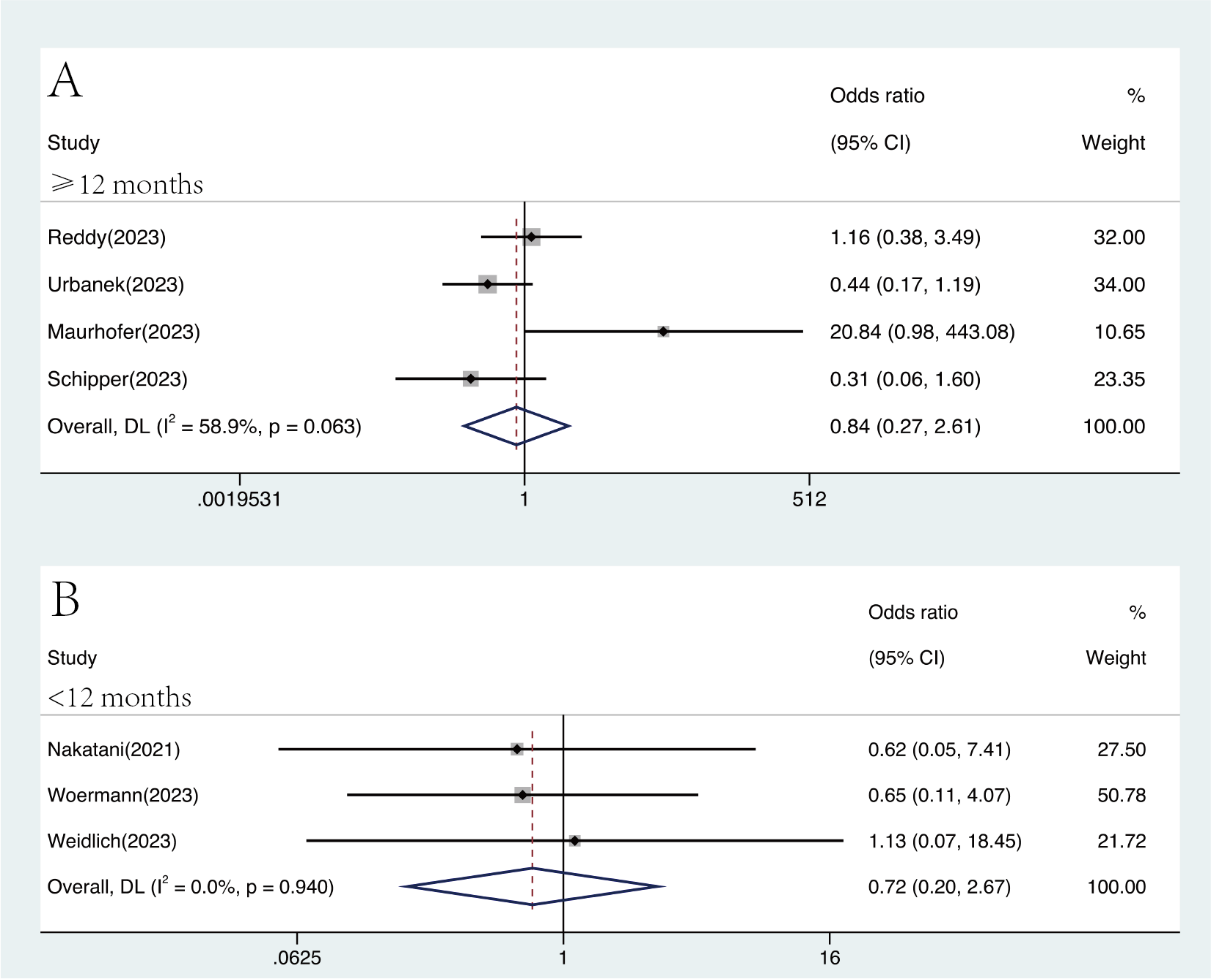


Abbreviation: CI = confidence interval, DL = DerSimonian and Laird approach.

**Supplementary Fig. 13:** Forest plots demonstrating the efficacy endpoint of atrial fibrillation patients for different ablation energy.
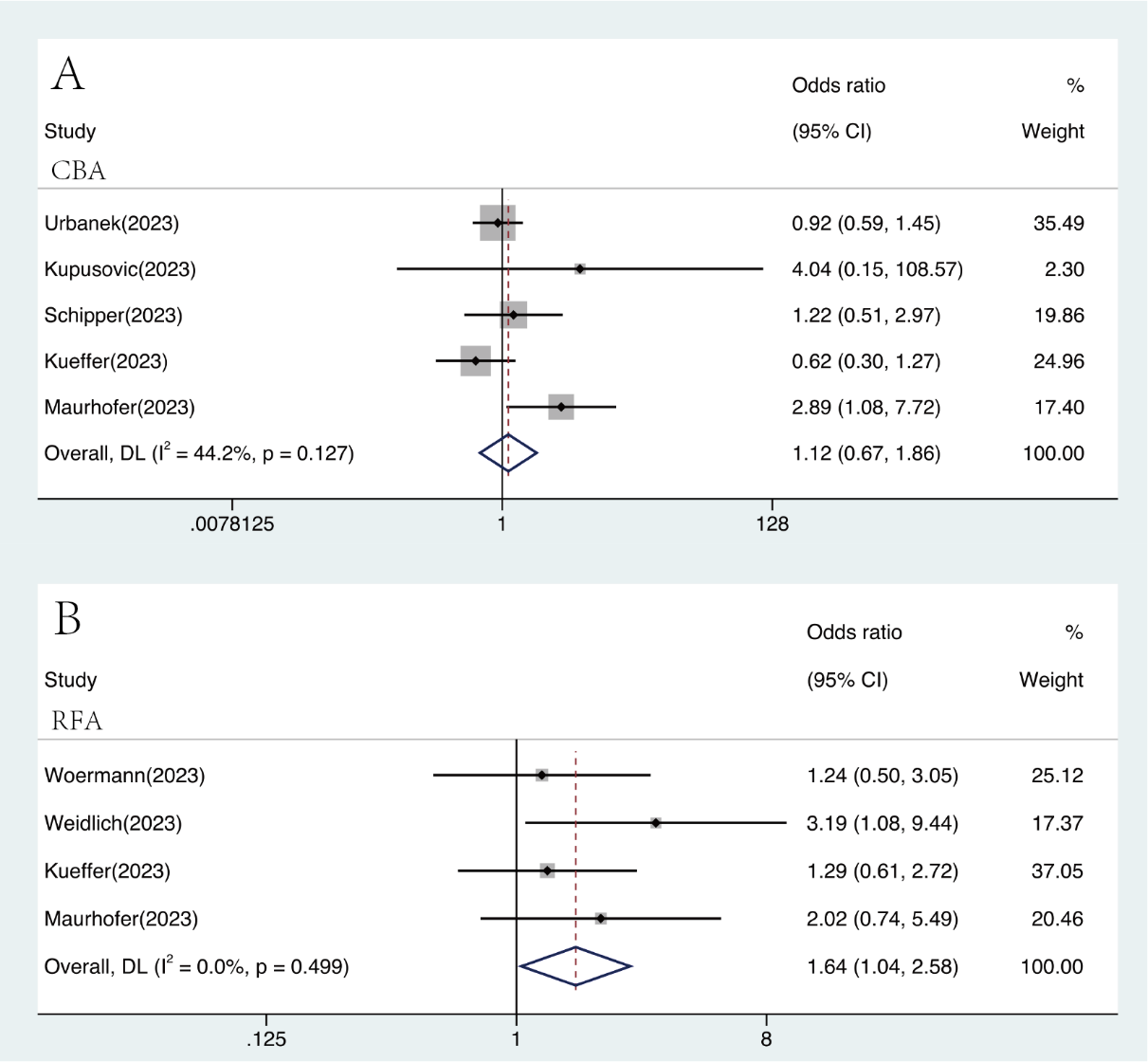


Abbreviation: CBA = cryoballoon ablation; RFA = radiofrequency ablation; CI = confidence interval, DL = DerSimonian and Laird approach.

**Supplementary Fig. 14:** Forest plots demonstrating the safety endpoint of atrial fibrillation patients for different ablation energy.


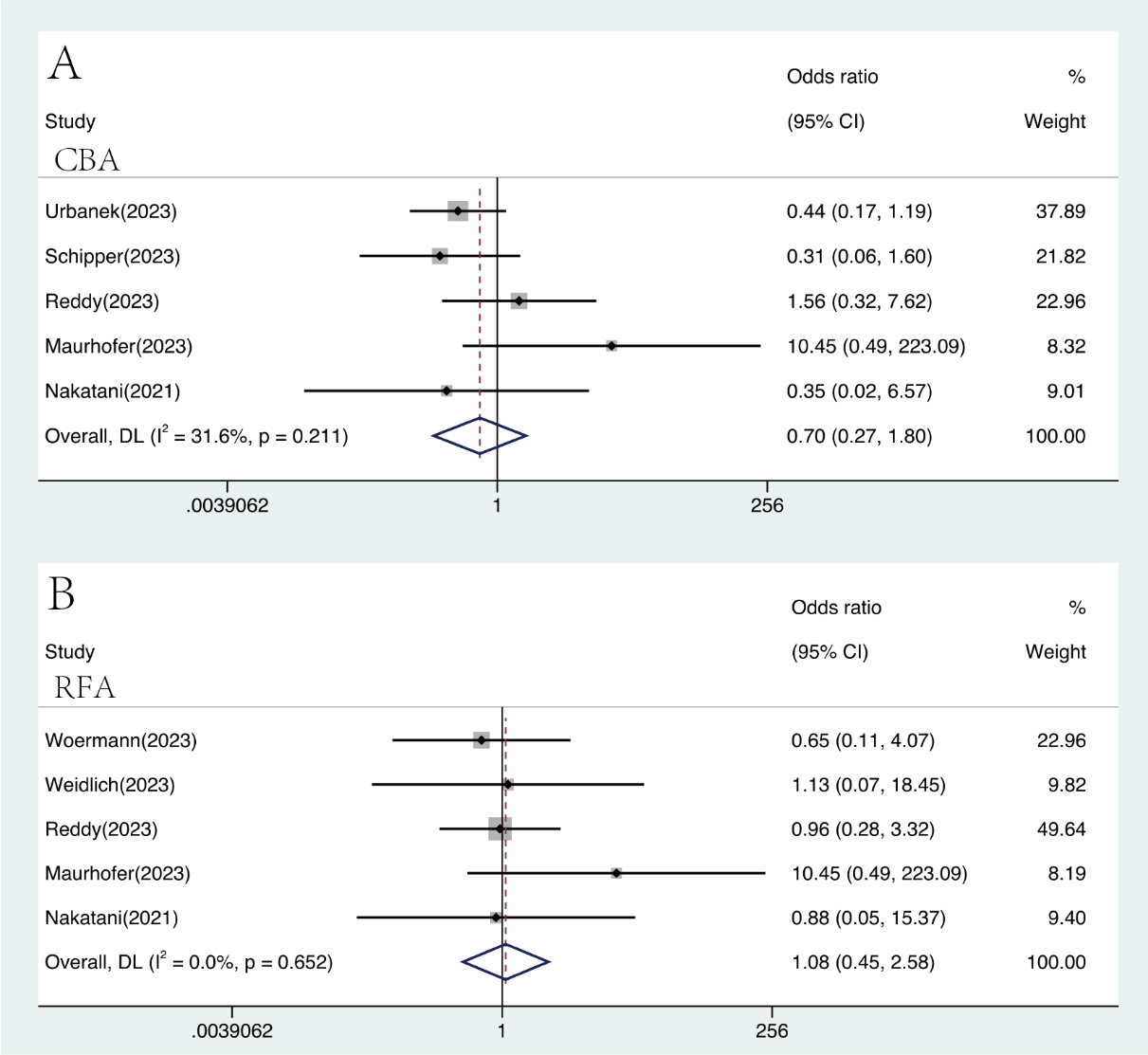


Abbreviation: CBA = cryoballoon ablation; RFA = radiofrequency ablation; CI = confidence interval, DL = DerSimonian and Laird approach.
