# Supplementary material for: Safety and Efficacy of Pulse Field Ablation in the Treatment of Atrial Fibrillation and Its Comparison with Traditional Thermal Ablation: A Systematic Review and Meta-Analysis
Source: Rev Cardiovasc Med. 2024 Nov 21;25(11):415. doi: 10.31083/j.rcm2511415 (PMC11607503; doi:10.31083/j.rcm2511415)
Supplement: Supplementary file 1 [file 2153-8174-25-11-415-s1.zip › Supplementary table(s).docx]

Page 2-4: **Supplementary Table 1:** PRISMA checklist.

Page 5-7: **Supplementary Table 2:** Search formula for each database.

Page 8: **Supplementary Table 3:** Methodological Index for Non-Randomized Studies (MINORS) criteria.

Page 9-11: **Supplementary Table 4:** Summary of the characteristics, procedures, and outcomes for all included studies which involved only pulsed field ablation as the ablation method.

**Supplementary Table 1:** PRISMA checklist.

| **Section and Topic** | **Item #** | **Checklist item** | **Location where item is reported** |
| --- | --- | --- | --- |
| **TITLE** | | |  |
| Title | 1 | Identify the report as a systematic review. | Page 1 |
| **ABSTRACT** | | |  |
| Abstract | 2 | See the PRISMA 2020 for Abstracts checklist. | N/A |
| **INTRODUCTION** | | |  |
| Rationale | 3 | Describe the rationale for the review in the context of existing knowledge. | Page 1 |
| Objectives | 4 | Provide an explicit statement of the objective(s) or question(s) the review addresses. | Page 1 |
| **METHODS** | | |  |
| Eligibility criteria | 5 | Specify the inclusion and exclusion criteria for the review and how studies were grouped for the syntheses. | Page 2 |
| Information sources | 6 | Specify all databases, registers, websites, organisations, reference lists and other sources searched or consulted to identify studies. Specify the date when each source was last searched or consulted. | Page 2 |
| Search strategy | 7 | Present the full search strategies for all databases, registers and websites, including any filters and limits used. | Page 2 |
| Selection process | 8 | Specify the methods used to decide whether a study met the inclusion criteria of the review, including how many reviewers screened each record and each report retrieved, whether they worked independently, and if applicable, details of automation tools used in the process. | Page 2 |
| Data collection process | 9 | Specify the methods used to collect data from reports, including how many reviewers collected data from each report, whether they worked independently, any processes for obtaining or confirming data from study investigators, and if applicable, details of automation tools used in the process. | Page 2 |
| Data items | 10a | List and define all outcomes for which data were sought. Specify whether all results that were compatible with each outcome domain in each study were sought (e.g. for all measures, time points, analyses), and if not, the methods used to decide which results to collect. | Page 2 |
|  | 10b | List and define all other variables for which data were sought (e.g. participant and intervention characteristics, funding sources). Describe any assumptions made about any missing or unclear information. | Page 2 |
| Study risk of bias assessment | 11 | Specify the methods used to assess risk of bias in the included studies, including details of the tool(s) used, how many reviewers assessed each study and whether they worked independently, and if applicable, details of automation tools used in the process. | Page 2 |
| Effect measures | 12 | Specify for each outcome the effect measure(s) (e.g. risk ratio, mean difference) used in the synthesis or presentation of results. | Page 5 |
| Synthesis methods | 13a | Describe the processes used to decide which studies were eligible for each synthesis (e.g. tabulating the study intervention characteristics and comparing against the planned groups for each synthesis (item #5)). | Page 5 |
|  | 13b | Describe any methods required to prepare the data for presentation or synthesis, such as handling of missing summary statistics, or data conversions. | Page 5 |
|  | 13c | Describe any methods used to tabulate or visually display results of individual studies and syntheses. | Page 5 |
|  | 13d | Describe any methods used to synthesize results and provide a rationale for the choice(s). If meta-analysis was performed, describe the model(s), method(s) to identify the presence and extent of statistical heterogeneity, and software package(s) used. | Page 5 |
|  | 13e | Describe any methods used to explore possible causes of heterogeneity among study results (e.g. subgroup analysis, meta-regression). | Page 5 |
|  | 13f | Describe any sensitivity analyses conducted to assess robustness of the synthesized results. | Page 5 |
| Reporting bias assessment | 14 | Describe any methods used to assess risk of bias due to missing results in a synthesis (arising from reporting biases). | Page 5 |
| Certainty assessment | 15 | Describe any methods used to assess certainty (or confidence) in the body of evidence for an outcome. | Page 5 |
| **RESULTS** | | |  |
| Study selection | 16a | Describe the results of the search and selection process, from the number of records identified in the search to the number of studies included in the review, ideally using a flow diagram. | Page 5 |
|  | 16b | Cite studies that might appear to meet the inclusion criteria, but which were excluded, and explain why they were excluded. | Page 5 |
| Study characteristics | 17 | Cite each included study and present its characteristics. | Page 5 |
| Risk of bias in studies | 18 | Present assessments of risk of bias for each included study. | Page 5-6 |
| Results of individual studies | 19 | For all outcomes, present, for each study: (a) summary statistics for each group (where appropriate) and (b) an effect estimates and its precision (e.g. confidence/credible interval), ideally using structured tables or plots. | Page 5-6 |
| Results of syntheses | 20a | For each synthesis, briefly summarise the characteristics and risk of bias among contributing studies. | Page 5-6 |
|  | 20b | Present results of all statistical syntheses conducted. If meta-analysis was done, present for each the summary estimate and its precision (e.g. confidence/credible interval) and measures of statistical heterogeneity. If comparing groups, describe the direction of the effect. | Page 5-6 |
|  | 20c | Present results of all investigations of possible causes of heterogeneity among study results. | Page 5-6 |
|  | 20d | Present results of all sensitivity analyses conducted to assess the robustness of the synthesized results. | Page 5-6 |
| Reporting biases | 21 | Present assessments of risk of bias due to missing results (arising from reporting biases) for each synthesis assessed. | Page 5-6 |
| Certainty of evidence | 22 | Present assessments of certainty (or confidence) in the body of evidence for each outcome assessed. | Page 5-6 |
| **DISCUSSION** | | |  |
| Discussion | 23a | Provide a general interpretation of the results in the context of other evidence. | Page 6-10 |
|  | 23b | Discuss any limitations of the evidence included in the review. | Page 6-10 |
|  | 23c | Discuss any limitations of the review processes used. | Page 6-10 |
|  | 23d | Discuss implications of the results for practice, policy, and future research. | Page 6-10 |
| **OTHER INFORMATION** | | |  |
| Registration and protocol | 24a | Provide registration information for the review, including register name and registration number, or state that the review was not registered. | Page 1-2 |
|  | 24b | Indicate where the review protocol can be accessed, or state that a protocol was not prepared. | Page 1-2 |
|  | 24c | Describe and explain any amendments to information provided at registration or in the protocol. | Page 1-2 |
| Support | 25 | Describe sources of financial or non-financial support for the review, and the role of the funders or sponsors in the review. | Page 11 |
| Competing interests | 26 | Declare any competing interests of review authors. | Page 11 |
| Availability of data, code and other materials | 27 | Report which of the following are publicly available and where they can be found: template data collection forms; data extracted from included studies; data used for all analyses; analytic code; any other materials used in the review. | Page 10 |

**Supplementary Table 2:** Search formula for each database.

| **Query** | **Results**  **(19 October, 2023)** |
| --- | --- |
| ***PubMed*** | |
| *((((((((((((((((((((((((((("Atrial Fibrillation"[Mesh]) OR (AF[Title/Abstract])) OR (Atrial Fibrillations[Title/Abstract])) OR (Fibrillation, Atrial[Title/Abstract])) OR (Fibrillations, Atrial[Title/Abstract])) OR (Auricular Fibrillation[Title/Abstract])) OR (Auricular Fibrillations[Title/Abstract])) OR (Fibrillation, Auricular[Title/Abstract])) OR (Fibrillations, Auricular[Title/Abstract])) OR (Persistent Atrial Fibrillation[Title/Abstract])) OR (Atrial Fibrillation, Persistent[Title/Abstract])) OR (Atrial Fibrillations, Persistent[Title/Abstract])) OR (Fibrillation, Persistent Atrial[Title/Abstract])) OR (Fibrillations, Persistent Atrial[Title/Abstract])) OR (Persistent Atrial Fibrillations[Title/Abstract])) OR (Familial Atrial Fibrillation[Title/Abstract])) OR (Atrial Fibrillation, Familial[Title/Abstract])) OR (Atrial Fibrillations, Familial[Title/Abstract])) OR (Familial Atrial Fibrillations[Title/Abstract])) OR (Fibrillation, Familial Atrial[Title/Abstract])) OR (Fibrillations, Familial Atrial[Title/Abstract])) OR (Paroxysmal Atrial Fibrillation[Title/Abstract])) OR (Atrial Fibrillation, Paroxysmal[Title/Abstract])) OR (Atrial Fibrillations, Paroxysmal[Title/Abstract])) OR (Fibrillation, Paroxysmal Atrial[Title/Abstract])) OR (Fibrillations, Paroxysmal Atrial[Title/Abstract])) OR (Paroxysmal Atrial Fibrillations[Title/Abstract])) AND (((((((((((((((((("Pulsed Radiofrequency Treatment"[Mesh]) OR "Electroporation Therapies"[Mesh]) OR (Pulsed field ablation[Title/Abstract])) OR (pulsed field electrical ablation[Title/Abstract])) OR (Pulsed Radiofrequency Treatments[Title/Abstract])) OR (Radiofrequency Treatment, Pulsed[Title/Abstract])) OR (Radiofrequency Treatments, Pulsed[Title/Abstract])) OR (Treatment, Pulsed Radiofrequency[Title/Abstract])) OR (Treatments, Pulsed Radiofrequency[Title/Abstract])) OR (Pulsed Radio Frequency Treatment[Title/Abstract])) OR (Therapies, Electroporation[Title/Abstract])) OR (Therapy, Electroporation[Title/Abstract])) OR (Electroporation Therapy[Title/Abstract])) OR (Therapeutic Use of Electroporation[Title/Abstract])) OR (Electroporation Therapeutic Uses[Title/Abstract])) OR (Electroporation Therapeutic Use[Title/Abstract])) OR (Therapeutic Use, Electroporation[Title/Abstract])) OR (Therapeutic Uses, Electroporation[Title/Abstract]))* | **171** |
| ***Web of Science*** | |
| *#1 (TS=(atrial fibrillation)) OR AB=(AF OR atrial fibrillation OR Atrial Fibrillations OR Fibrillation, Atrial OR Fibrillations, Atrial OR Auricular Fibrillation OR Auricular Fibrillations OR Fibrillation, Auricular OR Fibrillations, Auricular OR Persistent Atrial Fibrillation OR Atrial Fibrillation, Persistent OR Atrial Fibrillations, Persistent OR Fibrillation, Persistent Atrial OR Fibrillations, Persistent Atrial OR Persistent Atrial Fibrillations OR Familial Atrial Fibrillation OR Atrial Fibrillation, Familial OR Atrial Fibrillations, Familial OR Familial Atrial Fibrillations OR Fibrillation, Familial Atrial OR Fibrillations, Familial Atrial OR Paroxysmal Atrial Fibrillation OR Atrial Fibrillation, Paroxysmal OR Atrial Fibrillations, Paroxysmal OR Fibrillation, Paroxysmal Atrial OR Fibrillations, Paroxysmal Atrial OR Paroxysmal Atrial Fibrillations)*  *#2 (TS=(pulsed field ablation)) OR AB=(pulsed field ablation OR Pulsed Radiofrequency Treatment OR Pulsed Radiofrequency Treatments OR Radiofrequency Treatment, Pulsed OR Radiofrequency Treatments, Pulsed OR Treatment, Pulsed Radiofrequency OR Treatments, Pulsed Radiofrequency OR Pulsed Radio Frequency Treatment OR Electroporation Therapies OR Therapies, Electroporation OR Therapy, Electroporation OR Electroporation Therapy OR Therapeutic Use of Electroporation OR Electroporation Therapeutic Uses OR Electroporation Therapeutic Use OR Therapeutic Use, Electroporation OR Therapeutic Uses, Electroporation)*  *#3 (#1 AND #2)* | ***450*** |
| ***Embase*** | |
| *1(AF or atrial fibrillation or Atrial Fibrillations or Fibrillation, Atrial or Fibrillations, Atrial or Auricular Fibrillation or Auricular Fibrillations or Fibrillation, Auricular or Fibrillations, Auricular or Persistent Atrial Fibrillation or Atrial Fibrillation, Persistent or Atrial Fibrillations, Persistent or Fibrillation, Persistent Atrial or Fibrillations, Persistent Atrial or Persistent Atrial Fibrillations or Familial Atrial Fibrillation or Atrial Fibrillation, Familial or Atrial Fibrillations, Familial or Familial Atrial Fibrillations or Fibrillation, Familial Atrial or Fibrillations, Familial Atrial or Paroxysmal Atrial Fibrillation or Atrial Fibrillation, Paroxysmal or Atrial Fibrillations, Paroxysmal or Fibrillation, Paroxysmal Atrial or Fibrillations, Paroxysmal Atrial or Paroxysmal Atrial Fibrillations).ab.*  *2(pulsed field ablation or Pulsed Radiofrequency Treatment or Pulsed Radiofrequency Treatments or Radiofrequency Treatment, Pulsed or Radiofrequency Treatments, Pulsed or Treatment, Pulsed Radiofrequency or Treatments, Pulsed Radiofrequency or Pulsed Radio Frequency Treatment or Electroporation Therapies or Therapies, Electroporation or Therapy, Electroporation or Electroporation Therapy).ab.*  *3 1 and 2* | ***257*** |
| ***Total*** | ***878*** |
| ***Total without duplicates*** | ***540*** |

**Supplementary Table 3:** Methodological Index for Non-Randomized Studies (MINORS) criteria.

| Author | A clearly stated aim | Inclusion of consecutive patients | Prospective collection of data | Endpoints appropriate to the aim of the study | Unbiased assessment of the study endpoint | Follow-up period appropriate to the aim of the study | Loss to follow up less than 5% | Prospective calculation of the study size | An adequate control group | Contemporary groups | Baseline equivalence of groups | Adequate statistical analyses | MINORS score |
| --- | --- | --- | --- | --- | --- | --- | --- | --- | --- | --- | --- | --- | --- |
| Verma(2023) | ★ | ★ | ★ | ★ | ★ | ★ | ★ | ✩ |  |  |  |  | 7/8 |
| Turagam(2023) | ★ | ★ | ✩ | ★ | ★ | ★ | ★ | ✩ |  |  |  |  | 6/8 |
| Tilz(2023)-wide | ★ | ★ | ★ | ★ | ★ | ★ | ★ | ✩ | ★ | ★ | ★ | ★ | 11/12 |
| Tilz(2023)-pulsed | ★ | ★ | ★ | ★ | ★ | ★ | ★ | ✩ |  |  |  |  | 7/8 |
| Schmidt(2022) | ★ | ★ | ★ | ★ | ★ | ★ | ✩ | ✩ |  |  |  |  | 6/8 |
| Schmidt(2023) | ★ | ★ | ✩ | ★ | ★ | ★ | ★ | ✩ |  |  |  |  | 6/8 |
| Reddy(2019) | ★ | ★ | ★ | ★ | ★ | ★ | ✩ | ✩ |  |  |  |  | 6/8 |
| Reddy(2021) | ★ | ★ | ★ | ★ | ★ | ★ | ✩ | ✩ |  |  |  |  | 6/8 |
| Plank(2023) | ★ | ★ | ✩ | ★ | ★ | ★ | ★ | ✩ |  |  |  |  | 6/8 |
| Magni(2022) | ★ | ★ | ? | ★ | ★ | ★ | ✩ | ✩ |  |  |  |  | 6/8 |
| Lemoine(2022) | ★ | ★ | ★ | ★ | ★ | ★ | ✩ | ✩ |  |  |  |  | 6/8 |
| Gunawardene(2023) | ★ | ★ | ★ | ★ | ★ | ★ | ★ | ✩ | ★ | ★ | ★ | ★ | 11/12 |
| Fueting(2022) | ★ | ★ | ★ | ★ | ★ | ★ | ★ | ✩ |  |  |  |  | 7/8 |
| Duytschaever(2023) | ★ | ★ | ★ | ★ | ★ | ★ | ✩ | ✩ |  |  |  |  | 6/8 |
| Guo(2023) | ★ | ★ | ★ | ★ | ★ | ★ | ★ | ✩ |  |  |  |  | 7/8 |
| Davong(2023) | ★ | ★ | ★ | ★ | ★ | ★ | ✩ | ✩ |  |  |  |  | 6/8 |

Note: ★indicates 1 score, ✩indicates 0 score.

**Supplementary Table 4:** Summary of the characteristics, procedures, and outcomes for all included studies which involved only pulsed field ablation as the ablation method.

| Study | Study type | Participants size | | AF pattern | Follow-up | Total procedure duration | Fluoroscopy time | Freedom from AF recurrence | Total complications |
| --- | --- | --- | --- | --- | --- | --- | --- | --- | --- |
| Verma 2023 | Prospective cohert study | 300 | PAF  150 (50%) | | 12-month | 134 ± 50 for PAF  145 ± 60 for PSAF | 26 ± 17 for PAF  29 ± 21 for PSAF | 69.5% for PAF  62.3% for PSAF | 1(0.7%) for PAF  1(0.7%) for PSAF |
| Turagam 2023 | Retrospective cohert study | 1568 | PAF  1021(65%) | | 12-month | 61 (40-90) | 12(7-19) | 78.1% | 93(5.9%) |
| Tilz 2023 wide | Prospective cohert study | 30 | PAF  20 (67%) | | 12-month | 66(60-74) | 11(10-15) | 90% | 4(13%) |
| Tilz 2023 pulsed | Prospective cohert study | 50 | PAF  28(56%) | | 6-month | 27.6 ± 6.6 | 6 ± 2 | 82% | 3(6%) |
| Schmidt 2022 | Prospective cohert study | 191 | PAF  119 (62%) | | 6-month | 39 ± 14 | 9 ± 4 | 91% | 9(4.6%) |
| Schmidt 2023 | Retrospective cohert study | 1233 | PAF  742 (60%) | | 12-month | 50(40-87) | 14(9-21) | 74% | 45(3.6%) |
| Reddy 2019 | Prospective cohert study | 81 | PAF  17 (30%) | | 12-month | 92 ± 27.4 | 13.1 ± 7.6 | 87.4% | 1(1.2%) |
| Reddy  2021 | Prospective cohert study | 121 | PAF | | 12-month | 96.2±30.3 | 13.7±7.8 | 78.5% | 4(3.3%) |
| Plank  2023 | Retrospective cohert study | 231 | PAF  127(55%) | | 12-month | 38±16 | 8±4 | 74.2% | 8(3.5%) |
| Magni 2022 | Prospective cohert study | 100 | PAF  80(80%) | | 3-month | 60.9±26.8 | 13±7.9 | 92% | 2(2%) |
| Lemoine  2022 | Prospective cohert study | 138 | PAF  52(38%) | | 12-month | 78±22 | 16±7 | 90.4% for PAF  60.3% for PSAF | 5(3.6%) |
| Gunawardene 2023 | Prospective cohert study | 75 | PSAF | | 12-month | PVI+:91 ± 30  PVI: 76 ± 31 | PVI+:14 ± 7  PVI:14 ± 7 | 76.9% for PVI+  73% for PVI | 2(2.7%) |
| Fueting  2022 | Prospective cohert study | 30 | PAF | | 3-month | 116(103-129) | 26(20-30) | 93.3% | 1(3.3%) |
| Duytschaever 2023 | Prospective cohert study | 226 | PAF | | 12-month | WAVE1:82.4±20  WAVE2:70.1±27.7 | WAVE1:9.8±6.8  WAVE2:7.8±7.0 | 70.9% | 0(0%) |
| Guo 2023 | Prospective cohert study | 18 | PAF | | 8-month | 64.1±18.2 | 12.3±3.5 | 83% | 0(0%) |
| Davong  2023 | Prospective cohert study | 45 | PSAF | | 6-month | 84.1±20 | 23.6±6.8 | 80% | 3(6.6%) |

Abbreviation: AF: atrial fibrillation; PFA: Pulse field ablation; RFA: Radiofrequency ablation; CBA: Cryoballoon ablation; PAF: Paroxysmal atrial fibrillation; PSAF: persistent atrial fibrillation; PVI: pulmonary vein isolation
