# Supplementary figures and images for: Safety and Efficacy of Pulse Field Ablation in the Treatment of Atrial Fibrillation and Its Comparison with Traditional Thermal Ablation: A Systematic Review and Meta-Analysis
Source: Rev Cardiovasc Med. 2024 Nov 21;25(11):415. doi: 10.31083/j.rcm2511415 (PMC11607503; doi:10.31083/j.rcm2511415)

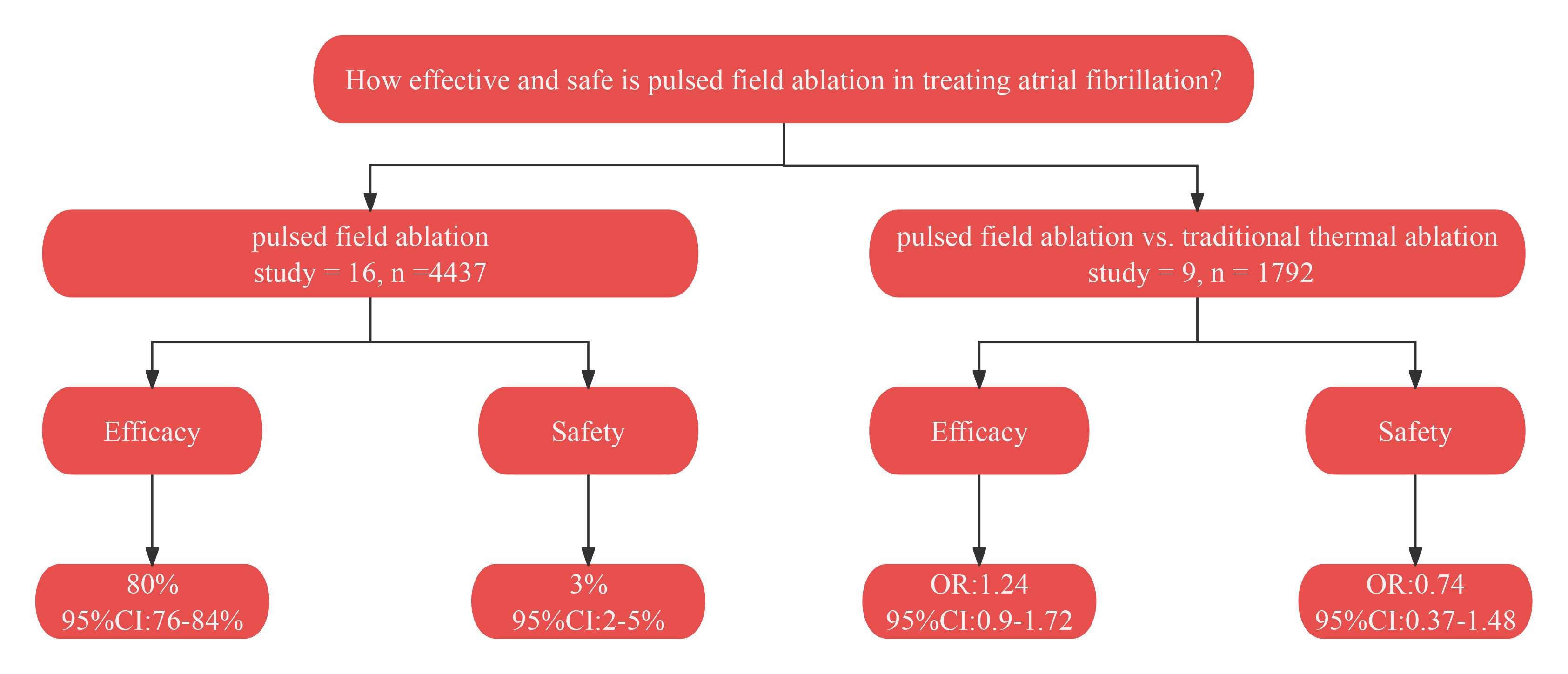

Supplement: Supplementary file 1 [file 2153-8174-25-11-415-s1.zip › Supplementary Material.tif]
